# Federated edge-AI for reliable and privacy-preserving pipeline leak detection in drone swarms using neutrosophic sugeno-weber norms

**DOI:** 10.1038/s41598-026-42794-4

**Published:** 2026-03-16

**Authors:** Rana Muhammad Zulqarnain, Muhammad Shazib Hameed, Ghulamullah Saeedi, Muntazim Abbas Hashmi, Eman Noreen, Muhammad Kamran, Vladimir Simic

**Affiliations:** 1 School of Business, Xian International University, Xian, 710077 Shaanxi China; 2 Institute of Mathematics, Khwaja Fareed University of Engineering & Information Technology, Rahim Yar Khan, 64200 Pakistan; 3 Department of Mathematics, Kabul Polytechnic University, Kabul, Afghanistan; 4 Department of Mathematics, Saveetha School of Engineering, Saveetha Institute of Medical and Technical Sciences, Saveetha University, Chennai, 602105 Tamil Nadu India; 5 Department of Computer Science and Engineering, Korea University, 145 Anam-ro, Seongbuk-gu, Seoul, 02841 Republic of Korea; 6 Faculty of Engineering, Dogus University, 34775 Umraniye, Istanbul Türkiye; 7 Department of Marine Navigation, Vilnius Gediminas Technical University, I. Kanto g. 7, Klaipèda, 92123 Lithuania

**Keywords:** Intuitionistic Neutrosophic Set, Sugeno-Weber, WASPAS, Decision Making, Gas pipelines, Leak detection, Engineering, Mathematics and computing

## Abstract

The ability to monitor the safety of natural gas pipelines is guaranteed by leak detection. Systems are capable of responding quickly, and very precisely to events because delays during such events can lead to serious environmental consequences, hurt, damage, or even danger. Federated is a special framework that exists in this work. Leak Detection of Natural Gas pipelines Edge-A-enabled autonomous drone swarms will be real-time, where smart drones will be able to cooperate and reduce latency, keep sensitive data, and improve detection of anomalies in dynamic operational conditions such as complex decentralized control. system also needs advanced systems of decision-making that are capable of dealing with uncertainty, shifting goals, and information gaps or ambiguous information. The research will, in an attempt to achieve this, emphasize the Multi-Criteria Decision-Making (MCDM) methods that have been used over the years as an alternative method of analysis, which is systematic and founded on alternative performance measures. The precedent versions of MCDM, which applied the theory of fuzzy set, allowed the analysts to convey their judgments with vagueness and partial truth. As uncertainty and conflicting decision environments increased, however, neutrosophic sets were included to describe the degree of truth, falsity and indeterminacy on their own. This was a later representation that was refined to describe hesitation more by using ambiguous membership and non-membership functions of intuitionistic fuzzy sets (IFS). The combined paradigms led to Intuitionistic Neutrosophic Set (INS), a paradigm of powerful mathematics that can reflect the complexity and ambiguity of the decision-making problems of the real world. In this research, the INS framework is used with Sugeno-Weber (SW) aggregation operators to come up with a hybrid DM framework that is optimally designed to respond to the real-time leaks detection and assessment in pipeline networks. The proposed INS-SW solution is contrasted with the time-tested approach to the evaluation of performance, the Weighted Aggregated Sum Product Assessment (WASPAS), as it is easy to operate and can generate credible ranks. The comparative outcomes indicate that the INS-SW model can be better adapted to uncertain, interdependent and dynamic operation environments and is more robust and precise in that case. In general, the results suggest that the suggested framework adds to the fact and veracity of the drone-based leakage detection to a substantial degree and can provide a scalable and intelligent decision-making tool related to the imperative energy infrastructure. Besides this application in specific, the paper would also be applicable in developing uncertainty-sensitive decision science further, besides offering an insight into how to develop sustainable, intelligent, and resilient energy systems in future industrial processes.

## Introduction

The smarter automation and decentralization age is changing industries into one, which is redefining itself through the application of advanced DM models^1^ and autonomous technologies^2^. Rather than being linear and deterministic as with the traditional methods, the modern-day industrial problems are open to adaptive frameworks and are able to deal with uncertainty, ambiguity, and dynamic information in an unpredictable and dynamic way. To satisfy these needs, the following paper suggests a hybrid DM framework, which is referred to as^3^ to be built with the aid of INS, which is referred to as^4^ and the SW aggregation functionality, which is referred to as^5^. Combined using these factors, they form a strong system of modeling vagueness, contradiction, and indeterminacy in MCDM environments^6^. The INS framework is based on the prospects of the classical set-based theories, such as FS, IFS, and NS^7^, and it adds three additional dimensions, i.e., truth, falsity, and indeterminacy, which allow representing uncertainty at a more sophisticated and accurate level. The model proves to be extremely efficient in DM settings where it is integrated with the nonlinear adaptability of SW operators^8^ in the sense that the criteria are mutually dependent and intricate. The proposed model will be benchmarked against the WASPAS^9^ approach to the evaluation of its performance against the recently established hybrid MCDM framework in terms of combining weighted sum and product models: the WASPAS approach, which is a widely recognized and utilized hybrid technique in terms of the combination of the weighted sum and product model. This comparison indicates the performance of each method when dealing with uncertainty, where there are many stakeholders and real-time decision needs. To illustrate the proposed framework practically, Federated Edge-AI Enabled Autonomous Drone Swarms in Real-Time Leak Detection in Natural Gas Pipelines is implemented using the proposed framework. Such swarms of drones are one application of the next-generation infrastructure monitoring systems whereby decentralized intelligence reduces the latency, data privacy, and DM can be scaled by adding more drones to the swarm. The problem of detecting the leakage of natural gas pipelines is a significant concern in terms of safety and the environment since despite the fact that even the slightest delay in the very process of its implementation may lead to tremendous losses, threats to human safety and the devastation of the environment. The problem with these systems is that they tend to be multidimensional and need to be considered concerning a great variety of variables, such as the accuracy of the detection, the amount of energy that is consumed, the reliability of the communication, and the impact on the environment. Moreover, the interplay of various stakeholders, such as engineers, controlling agencies, and manufacturing companies introduce conflicting interests and subjective perceptions and considerations that are difficult to capture using traditional models. This issue can be addressed using the proposed INS-SW framework because it is a flexible and uncertainty-aware framework, which would be in a position to accurately reflect the realities in the modern decision settings. The advantages of being an adaptable, accurate and interpretable intelligent DM can be substantiated by its comparison to WASPAS. Besides the theoretical developments, the study offers practical developments in the scalable and effective system of decision support, which may be applied in smart energy systems^10^, autonomous robotics^11^ and resilient infrastructure^12^. The research will assist in improving the performance of industries and increase the reliability of the process of decision-making by inventing the methodologies to involve the uncertainties, sustainability, and complexity to manage the critical infrastructure which will make the research stronger and more credible in its implications on the changes that need to be made to the approach to research and practice of the process of managing the infrastructure under the condition of critical conditions^13^ in the first place.

### **review of literature**

The increasing complexity of the modern systems and the necessity to act in the face of uncertainty, ambiguity and conflicting information have had an impact on smart DM framework development^14^. When vague or imprecise data appeared, it could not be modeled by previous paradigms such as classical binary logic, which was developed by^15^ and crisp set theory, which was developed by^16^, and thus gave rise to fuzzy set theory^17^. Membership degrees were introduced in the interval [0,1] and fuzzy sets provided a more relaxed approach to the partial truth, making it possible to make substantial advancements in the fields of control systems, pattern recognition and decision science^18^. It is based on this that the representational power of fuzzy systems with membership and non-membership degrees^20^ is added as well as a hesitation margin to reveal uncertainty as employed by IFS^19^ to represent uncertainty. It was a more sophisticated modeling form, which allowed finer-tuned judgments where data were incomplete or subjective. To improve group DM and manage divergent expert opinion, scientists also developed further on aggregation operators^21^ and preference models. Subsequently, a proposal of the Pythagorean fuzzy sets^22^ was presented, whereby the squared sum of the degree of membership and non-membership could fall in the unity, as suggested by other scholars, as noted in^23^. Such systems have been very useful in the unpredictable area of medical diagnostics, resources, and risk assessment, where cross entropy tools^24^ and divergence tools^25^ have been adopted to enhance accuracy of rankings. Another important conceptual breakthrough was achieved in NS^26^ that described the truth, falsity, and indeterminacy as three independent dimensions; it was providing a more generalized way of dealing with inconsistent or incomplete information. The model was later extended to interval-valued NS^27^, NS of having uncertainty that is represented by intervals of values to be more realistic in situations of DM processes. At the same time, operators of parallel aggregation, such as the SW operator, gained popularity due to the ability of the operator to describe nonlinear relationships and interactive behavior of criteria. Combining these operators with the INS, the resulting DM model is strong and flexible and has the ability to address dynamically interacting attributes and complex uncertain structures. WASPAS is among the oldest multi-criteria DM methods, yet the method is still popular because it is efficient in computation, interpretation, and reliable ranking. But it suffers performance in an environment where it is characterized by either fuzzy judgment, subjective preferences, or a high degree of estimations. In such circumstances, under these conditions, the models based on the INS are better than those based on WASPAS and present more comprehensive and robust evaluations in the atmosphere of uncertainty and contradiction. These theoretical developments of this paper are applied in a strict real-world example Federated Edge-AI Enabled Autonomous Drone Swarms by Real-Time Leak Detection in Natural Gas Pipelines. These drone swarms are decentrally intelligent on the board to process sensor data, and nevertheless they communicate in order to achieve low latency, privacy preservation, and scalability. The pressure to detect leakages in natural gas infrastructure is a high-stakes issue whose delay has a devastating effect on the environment and safety, as well as financial losses. In this instance, successful DM requires localization of numerous dependent criteria such as accuracy of detection, energy efficiency, communication viability, and environmental sustainability. The fact that INS is combined with the SW aggregation operator implies that this research presents a full and uncertainty-sensitive framework that has the potential of combining numerous sensor data, removing conflicting data, and providing real-time and reliable DM under the conditions of complex and risk-averse operations. Recent research has used hypersoft sets^28^, NS for medical diagnosis^29^, q-rung picture sets for supply chain^30^, picture fuzzy soft for banking selection^31^, and a hybrid q-rung picture fuzzy DM strategy^32^ to achieve remarkable results.

### Motivations of the article

Real-time leakage detection of natural gas pipelines^33^ is a critical component of industrial safety that is directly related to environmental protection, efficiency of operation, and human well-being. Leak detection is also a very complex process, although it is a critical process. Uncertainties, ambiguity, and indecisiveness are caused by the constrained capability of the sensors, distortion of signals, and the biases that are of human nature. Such issues lower the integrity of classical or traditional probabilistic approaches to DM that are not meant to effectively deal with uncertainty. This drawback highlights the fact that there is a need to possess some form of computational model that can be trusted to model and compute ambiguous options with a more precise account. Using INS-SW is one of the solutions that could be applied. The sensor data in a real-field system is hardly ever complete and precise: they might be inconclusive, the drone-like inspection can be subject to errors, and various indications can overlap very simply in a case of faults of any sort. The simplification or neglecting of such uncertainty in the use of classical models will increase the likelihood of making misinterpretations and errors in detection. Conversely, the individual development of the $IN_{SW}$ system is designed to capture and deal with imprecision to achieve more reliable and robust DM in complicated detection conditions. INS captures the truth (evidence to support a leak), falsity (evidence to reject a leak), and indeterminacy (uncertain or conflicting information), by which a more reflective and realistic picture of its operational data is provided. The other reason why this study was conducted is that industrial monitoring systems are becoming highly complex. Due to the use of autonomous swarms of drones^34^, edge-level computation, and multi-modal sensing, operators now need to make sense of large volumes of heterogeneous data. Despite the fact that this improves coverage and accuracy of detection, conflicts, redundancy, and discrepancy are created. Such situations cannot do without MCDM frameworks. The $IN_{SW}$ operator, being an advanced form of MCDM, is an operator that is well suited to the optimization process of the decision in uncertain and data-intensive environments. Errors of leak detection, such as false alarms or false negatives, are dangerous and can lead to environmental degradation and safety losses. Most of these mistakes are brought about by the misunderstanding and discrepancy in interpreting sensor evidence. Detection systems can mitigate these risks greatly by combining the use of $IN_{SW}$ based computational algorithms to provide the operators with transparent, interpretable, and trustworthy decision support. Neutrosophic systems^35^ are of the form where uncertainty is explicitly posed, unlike the conventional black-box models, which improve the interpretability and accountability of automated DM processes. Lastly, motivation goes to the construction of smart infrastructure and renewable energy systems. Distribution of monitoring environments with differences in terrain, climatic conditions, and sensor performance increases uncertainty in pipeline surveillance. The INS-SW-based DM structures offer a standardized and resilient solution, which is in line with the current rise of stability, resilience, and sustainability requirements of energy networks. To conclude, the significance of the current study is the necessity to integrate uncertainty into autonomous leak detection systems. The given approach, which is based on the INS and SW operators, will overcome the drawbacks of the traditional methods, increase the quality of decisions, minimize the possibility of detecting anomalies, and improve the stability of intelligent energy systems. A comparative evaluation to WASPAS further highlights the benefits that are reported on the use of the tool $IN_{SW}$ in managing ambiguity, which justifies its future-ready and uncertainty-sensitive ability as a solution to leak detection in real-time.

### Objective of the article

The goals of the study Federated Edge-AI Enabled Autonomous Drone Swarms for Real-Time Leak Detection in Natural Gas Pipelines are as follows:

- To establish the theoretical foundations of INS within fuzzy and neutrosophic frameworks, defining their mathematical structure and demonstrating their effectiveness in modeling uncertainty, contradiction, and ambiguity in DM.
- To design and refine INS-based aggregation operators, similarity measures, and inference mechanisms such as those derived from SW-norms^36^ capable of capturing nonlinear dependencies and improving the robustness of MCDM.
- To establish scalable computational algorithms and decision models founded on INS and make them understandable, computationally efficient and transparent to complex real-time industrial problems. item To use the INS-SW framework in the case study (federated edge-enabled autonomous drone swarms) to detect natural gas pipeline leaks in real-time, it is necessary to integrate heterogeneous sensor data (gas concentration, thermal, acoustic, and geospatial data) into consistent, reliable and explanable decision products. item To carry out comparative analysis of INS based models with IFS, classical NS and recognized MCDM techniques like WASPAS to evaluate computational performance, versatility, and ranking accuracy in the presence of uncertainty.
- To extend the applicability of INS-driven frameworks by exploring hybrid integrations with soft computing techniques (e.g., neural networks, genetic algorithms, and rough sets) and validating their potential across broader domains such as environmental monitoring, sustainable infrastructure, and industrial safety.

### Contributions to the article

The article under discussion, Federated Edge-AI Enabled Autonomous Drone Swarms in Natural Gas Pipeline Real-Time Leak Detection, also contributes some useful aspects to the issue of uncertainty and complexity in the distributed monitoring systems:

- To implement a computational model of INS to handle uncertainty, indeterminacy, and inconsistency of multi-source sensor information, thus enhancing the accuracy and responsiveness of leak detection in natural gas pipelines by autonomous drone swarms.
- To develop a federated INS-based DM model to incorporate heterogeneous sensor data in terms of thermal data, gas concentration levels, acoustic signals, and geostatic data that can facilitate robust, scalable, and adaptable localization of leaks in dynamic and uncertain environmental conditions.
- To optimize multi-criteria group DM by aligning conflicting results among distributed drones and high-risk areas and to make sure that the final decision would be based on the sensor-level, as well as swarm-level, evidence.
- To illustrate that the INS framework outperforms classical fuzzy and probabilistic frameworks due to their explicit modeling of truth, falsity, and hesitation, which enables drones to use incomplete or contradictory information and provide explainable and accurate decision results.
- To enhance the development of interpretable and autonomous monitoring schemes that can operate effectively in large-scale and convoluted infrastructures where it is infeasible or costly to manage the centralized DM.
- To further demonstrate the applicability of INS and its generalizability to other applications that need distributed DM in the presence of uncertainty, such as environmental surveillance, critical infrastructure control, and surveillance systems with safety requirements.

The given study presents an INS based Sugeno-Weber aggregation model that is essentially different to the present-day INS-based MCDM methods like INS-TOPSIS, INS-WASPAS, and INS-EDAS. Contrary to the linear or compensatory aggregation schemes that are popular in the literature^37^, the given framework utilizes the nonlinear Sugeno-Weber norm to simulate the interaction effects of the degrees of truth, indeterminacy and falsity. The nonlinear behavior can be better aggregated using conflicting and uncertain information which is a hallmark of drone-based pipeline monitoring environment. In addition, the suggested operators are designed to meet the distributed decision fusion with federated environments, which are applicable to privacy-preserving and real-time applications, which are not explicitly considered in the current INS-SW formulations. Although the title and the abstract focus on the idea of federated edge-AI and drone swarms, the main methodological value of this work is the creation of a new type of SW-based aggregation operators with INS-SW. Such operators are explicitly developed to deal with the uncertainty, incompleteness, and conflict in distributed sensor data- features that are exaggerated in federated drone-based monitoring systems. The so-called federated edge-AI will be the mode of operation the INS-SW model is implemented: the drones are used as edge nodes, which locally transform sensor data into INS triplets, and the means of aggregation is achieved using only aggregated INS matrices, without direct data transmission.

### Organization of the article

In order to establish a strong theoretical foundation while offering practical insights for optimizing uncertain DM in real-time pipeline surveillance, this study is organized into a coherent sequence of sections:

- Argues about existing methodologies of natural gas pipeline leak detection that they lack the capability to handle ambiguous, contradictory, or missing sensor data. This argument brings out the justification of the application of an INS-based approach in an effort to represent the uncertainty and indeterminacy in distributed settings. item suggests the formal mathematical notation of INS, the key concepts of the model, the key properties of the model, the advantages of the model over the conventional uncertainty models, that is fuzzy sets and classical probability theory, thus gives a solid theoretical foundation to the further analysis. item Varying the suggested DM methodology, the formation of decision matrices, the creation of the aggregation operators, and optimization approaches to integrate multi-modal sensors data delivered by self-driving fleets of drones at the federated edge.
- A case study on federated edge-intelligent drones performing real-time natural gas pipeline surveillance. As shown in this case study, the INS framework can be used to improve leak detection performance through the resolution of data ambiguity, the enhancement of localization accuracy, and adaptive collaboration at the swarm level.
- presents experimental outcomes and comparative research, emphasizing the precision, adaptability, and strength of the proposed model in comparison with traditional DM techniques, thus confirming its usefulness in practical application.
- The INS-SW framework is introduced that enhances autonomous drone-based leak detection through an effective management of uncertainty, ambiguity, and conflicting data. This combination method is also very effective in the monitoring of intelligent infrastructure, making the process more accurate, flexible, and reliable.
- Concludes the paper with an overview of the main contributions and future studies to create explainable, decentralized, and uncertainty-aware models of DM to further enhance resilient and scalable autonomous systems.

## Preliminaries

In this part, the fundamental ideas and mathematical model needed to exploit INS in the autonomous drone-based detection of leaks are provided. It begins by providing the summary of NS and then INS to show that these models are successful in bringing out the independent presence of truth, indeterminacy, and falsity properties, which are indeterminable during a conventional fuzzy or probabilistic model. Particular attention is paid to such basic steps of INS as the scoring and accuracy functions that are essential in the comparison and evaluation of alternative detection outcomes. Such inherent processes form the basis of extending the concept of INS to more complex MCDM systems in which the problems of non-homogeneous, uncertain, and dynamically evolving sensor data as captured by federated edge-based drone swarms in real-time pipeline monitoring occur.

### Definition 2.1

Let X be the universal set; the NS A in X is characterized by three membership functions

$$\begin{aligned} A=\{x,\tau _A(x),\iota _A(x),\phi _A(x):~x\in X\} \end{aligned}$$

where:

$\tau _A(x):X\rightarrow [0,1]$ represent the truth membership degree of the element *x* in *A*.

$\iota _A(x):X\rightarrow [0,1]$ represent the indeterminacy membership degree of element *x* in *A*.

$\phi _A(x):X\rightarrow [0,1]$ represents the falsity membership degree of an element *x* in *A*.

The condition is:

$$\begin{aligned} 0\le \tau _A(x),\iota _A(x),\phi _A(x)\le 1,~\forall ~x\in X \end{aligned}$$

In contrast to both classical sets and FS, in the case of NS, the three functions can be independent, i.e.,

$$\begin{aligned} 0\le \tau _A(x)+\iota _A(x)+\phi _A(x)\le 3 \end{aligned}$$

Such yielded elasticity allows NSs to portray uncertainty, indecisiveness, inconsistency, and incomplete information; thus, they are very well applicable to interrelated complicated situation-like problems like medical diagnosis.

### Definition 2.2

Consider the set X to be universal. The definition of an IFS A in X is :

$$\begin{aligned} A=\{x,\mu _A(x),\nu _A(x):x\in X\} \end{aligned}$$

where:

$\mu _A(x):X\rightarrow [0,1]$ represent the degree of membership of the element *x* in *A*.

$\nu _A(x):X\rightarrow [0,1]$ represent the degree of non-membership element *x* in *A*.

These values must satisfy the following condition:

$$\begin{aligned} 0\le \mu _A(x)+\nu _A(x) \le 1:~\forall ~ x\in X \end{aligned}$$

### Definition 2.3

Assume X is a random set. The following criteria determine whether a set A in X is an INS in X:

$$\begin{aligned} A=\{x,\tau _A(x),\iota _A(x),\phi _A(x):x\in X\} \end{aligned}$$

where: $\tau _A(x):X\rightarrow [0,1]$ represents the degree of truth membership of element x in A, $\iota _A(x):X\rightarrow [0,1]$ represents the degree of indeterminacy membership of element x in A, and $\phi _A(x):X\rightarrow [0,1]$ represents the degree of falsity membership of element x in A. With the condition:

$$\begin{aligned} 0\le \tau _A(x)+\phi _A(x)\le 1 \end{aligned}$$

$$\begin{aligned} 0\le \tau _A(x)+\iota _A(x)+\phi _A(x)\le 2 \end{aligned}$$

### Definition 2.4

Suppose $\alpha _{\sigma }= \Big \{{\tau },{\iota },{\phi }\Big \}$ and $\beta _{\sigma }= \Big \{{\tau },{\iota },{\phi }\Big \}$ be the *INS*s and $\lambda \ge 0$ then the following operation is defined as:

1. $$\alpha _{\sigma }\subseteq \beta _{\sigma } \Leftrightarrow \tau _{\alpha _{\sigma }} \le \tau _{\beta _{\sigma }},\; \iota _{\alpha _{\sigma }} \ge \iota _{\beta _{\sigma }},\; \phi _{\alpha _{\sigma }} \ge \phi _{\beta _{\sigma }}.$$
2. $$\alpha _{\sigma }= \beta _{\sigma }\Leftrightarrow \alpha _{\sigma }\supseteq \beta _{\sigma }$$ and $$\alpha _{\sigma }\subseteq \beta _{\sigma }$$.
3. $$\alpha _{\sigma }\cup \beta _{\sigma } = \Big \{ \tau _{\alpha _{\sigma }} \vee \tau _{\beta _{\sigma }},\; \iota _{\alpha _{\sigma }} \wedge \iota _{\beta _{\sigma }},\; \phi _{\alpha _{\sigma }} \wedge \phi _{\beta _{\sigma }} \Big \}.$$
4. $$\alpha _{\sigma }\cap \beta _{\sigma }=\Big \{ {\tau }_{\alpha _{\sigma }}\wedge {\tau }_{\beta _{\sigma }},\iota _{\alpha _{\sigma }}\vee \iota _{\beta _{\sigma }},\phi _{\alpha _{\sigma }}\vee \phi _{\beta _{\sigma }}\Big \}$$.
5. $$(\alpha _{\sigma })^{c}=\Big \{{\tau }_{\alpha _{\sigma }},{\iota }_{\alpha _{\sigma }},{\phi }_{\alpha _{\sigma }}\Big \}^{c}=\Big \{{\phi }_{\alpha _{\sigma }},{\iota }_{\alpha _{\sigma }},{\tau }_{\alpha _{\sigma }}\Big \}$$.
6. $$\alpha _{\sigma }\oplus \beta _{\sigma }=\left\{ {\tau }_{\alpha _{\sigma }}+ {\tau }_{\beta _{\sigma }}-{\tau }_{\alpha _{\sigma }}{\tau }_{\beta _{\sigma }},\iota _{\alpha _{\sigma }}\iota _{\beta _{\sigma }},\phi _{\alpha _{\sigma }}\phi _{\beta _{\sigma }}\right\}$$.
7. $$\alpha _{\sigma }\otimes \beta _{\sigma }=\left\{ {\tau }_{\alpha _{\sigma }}{\tau }_{\beta _{\sigma }},\iota _{\alpha _{\sigma }}+\iota _{\beta _{\sigma }}-\iota _{\alpha _{\sigma }}\iota _{\beta _{\sigma }}, \phi _{\alpha _{\sigma }}+ \phi _{\beta _{\sigma }}-\phi _{\alpha _{\sigma }}\phi _{\beta _{\sigma }}\right\}$$.
8. $$\lambda \alpha _{\sigma } = \left\{ 1 - (1 - \tau _{\alpha _{\sigma }})^{\lambda },\; \iota _{\alpha _{\sigma }}^{\lambda },\; \phi _{\alpha _{\sigma }}^{\lambda } \right\} .$$
9. $$(\alpha _{\sigma })^{\lambda }=\left\{ {\tau }_{\alpha _{\sigma }}^{\lambda },\{1-(1-\phi _{\alpha _{\sigma }})^{\lambda },{1-(1-\phi _{\alpha _{\sigma }})^{\lambda }}\right\}$$.

### Definition 2.5

^38^ To compare *INS* $\alpha _{\sigma }= \Big ({\tau },{\iota },{\phi }\Big )$ we introduced the score function as

$$\begin{aligned} \psi (\alpha _{\sigma })=\frac{2+\tau -\iota -\phi }{3} \end{aligned}$$

$$\psi (\alpha _{\sigma })\in [0,1]$$

if $\psi (\alpha _{\sigma })=\psi (\alpha _{\sigma }^c)$ then calculate the accuracy function

$$\begin{aligned} \mathcal {A}(\alpha _{\sigma })=\tau _{\alpha _{\sigma }}-\phi _{\alpha _{\sigma }} \end{aligned}$$

### Definition 2.6

The t-N and t-CN^38^ of SW operator is defined as

$$T_{SW}^{\gimel }(\alpha ,\beta )=\left\{ \begin{array}{ll} T_{\widetilde{{D}}}(\alpha ,\beta ), & ~ {\text { if}} ~ \gimel = -1,\\ max\left( 0,\frac{\alpha +\beta -1+\gimel \alpha \beta }{1+\gimel }\right) , & ~ {\text {if}} ~ -1< \gimel < +\infty ,\\ T_{\widetilde{P}}(\alpha ,\beta ), & ~ {\text {if}} ~ \gimel = +\infty ,\\ \end{array} \right.$$

where $T_{\widetilde{D}}(\alpha ,\beta )$ denotes the drastic t-N and $T_{\widetilde{P}}(\alpha ,\beta )$ denotes the algebraic product.

$$S_{SW}^{\gimel }(\alpha ,\beta )=\left\{ \begin{array}{ll} S_{\widetilde{{D}}}(\alpha ,\beta ), & ~ {\text { if}} ~ \gimel = -1,\\ min\left( 1,\alpha +\beta -\frac{\gimel }{1+\gimel }\alpha \beta \right) , & ~ {\text {if}} ~ -1< \gimel < +\infty ,\\ S_{\widetilde{P}}(\alpha ,\beta ), & ~ {\text {if}} ~ \gimel = +\infty ,\\ \end{array} \right.$$

where $S_{\widetilde{D}}(\alpha ,\beta )$ denotes the drastic t-CNs and $S_{\widetilde{P}}(\alpha ,\beta )$ denotes the algebraic sum.

##  Sugeno-Weber operator based on intuitionistic neutrosophic set

This section demonstrates the core operations of INS by employing the SW norm to significantly enhance data aggregation within autonomous drone swarms tasked with real-time leak detection in natural gas pipelines by using SW norms. The advanced aggregation model can easily address the complexities and uncertainty that can occur in the distributed, federated Edge-AI model that results in more precise and more timely pipeline leak detection as compared to the conventional methods.

$$\alpha _{\sigma } \bigoplus \beta _{\sigma }=\left( \Big (S_{SW}^{\gimel }({\tau _{\alpha _{\sigma }}},{\tau }_{\beta _{\sigma }})\Big ), \Big (T_{SW}^{\gimel }({\iota }_{\alpha _{\sigma }},{\iota }_{\beta _{\sigma }}),\Big (T_{SW}^{\gimel }({\phi }_{\alpha _{\sigma }},{\phi }_{\beta _{\sigma }})\Big )\right)$$

$$\alpha _{\sigma } \bigotimes \beta _{\sigma }=\left( \Big (T_{SW}^{\gimel }({\tau }_{\alpha _{\sigma }},{\tau }_{\beta _{\sigma }})\Big ), \Big (T_{SW}^{\gimel }({\iota }_{\alpha _{\sigma }},{\iota }_{\beta _{\sigma }}),\Big (S_{SW}^{\gimel }({\phi }_{\alpha _{\sigma }},{\phi }_{\beta _{\sigma }})\Big )\right)$$

### Definition 3.1

Let $\alpha _{\sigma }= \Big \{{\tau }_{\alpha _{\sigma }},{\iota }_{\alpha _{\sigma }},{\phi }_{\alpha _{\sigma }}\Big \}$ and $\beta _{\sigma }= \Big \{{\tau }_{\beta _{\sigma }},{\iota }_{\beta _{\sigma }},{\phi }_{\beta _{\sigma }}\Big \}$ are two *INS*s then some primeval operation of *INS*s on the basis of SW are given as

1. $$\alpha _{\sigma }\oplus \beta _{\sigma }= \left\{ \begin{array}{c} \Bigg ( {\tau }_{\alpha _{\sigma }} + {\tau }_{\beta _{\sigma }} - \frac{\gimel }{1+\gimel } {\tau }_{\alpha _{\sigma }} {\tau }_{\beta _{\sigma }}, \frac{{\iota }_{\alpha _{\sigma }} + {\iota }_{\beta _{\sigma }} - 1 + \gimel {\iota }_{\alpha _{\sigma }} {\iota }_{\beta _{\sigma }}}{1+\gimel }, \frac{{\phi }_{\alpha _{\sigma }} + {\phi }_{\beta _{\sigma }} - 1 + \gimel {\phi }_{\alpha _{\sigma }} {\phi }_{\beta _{\sigma }}}{1+\gimel }\Bigg ) \end{array} \right\} .$$
2. $$\alpha _{\sigma }\otimes \beta _{\sigma }= \left\{ \begin{array}{c} \Bigg (\frac{{\tau }_{\alpha _{\sigma }}+{\tau }_{\beta _{\sigma }}-1+\gimel {\tau }_{\alpha _{\sigma }}{\tau }_{\beta _{\sigma }}}{1+\gimel }, \frac{{\iota }_{\alpha _{\sigma }}+{\iota }_{\beta _{\sigma }}-1+\gimel {\iota }_{\alpha _{\sigma }}{\iota }_{\beta _{\sigma }}}{1+\gimel }, {{\phi }_{\alpha _{\sigma }}+{\phi }_{\beta _{\sigma }}-\frac{\gimel }{1+\gimel }{\phi }_{\alpha _{\sigma }}{\phi }_{\beta _{\sigma }}}\Bigg ) \end{array} \right\} .$$
3. $$\lambda \odot \alpha _{\sigma }= \left\{ \begin{array}{c} \Bigg ({\frac{1+\gimel }{\gimel }(1-(1-{\tau }_{\alpha _{\sigma }}(\frac{\gimel }{1+\gimel }))^{\lambda })}, {((1+\gimel )(\frac{\gimel {\iota }_{\alpha _{\sigma }}+1}{1+\gimel })^{\lambda }-1)\frac{1}{\gimel }}, {((1+\gimel )(\frac{\gimel {\phi }_{\alpha _{\sigma }}+1}{1+\gimel })^{\lambda }-1)\frac{1}{\gimel }}\Bigg ) \end{array} \right\} .$$
4. $$\alpha _{\sigma }^{\lambda }= \left\{ \begin{array}{c} \Bigg ({\frac{1}{\gimel }((1+\gimel )(\frac{\gimel {\tau }_{\alpha _{\sigma +1}}}{1+\gimel })^{\lambda }-1), \frac{1+\gimel }{\gimel }(1-(1-{\iota }_{\alpha _{\sigma }}(\frac{\gimel }{1+\gimel }))^{\lambda })}, \frac{1+\gimel }{\gimel }(1-(1-{\phi }_{\alpha _{\sigma }}(\frac{\gimel }{1+\gimel }))^{\lambda })\bigg ) \end{array} \right\}$$.

The proposed system consists of drones that are intelligent in the form of edge nodes and have gas concentration, pressure, thermal, and vibration sensors. They are then converted into information of intuitionistic neutrosophic form by identifying prediction confidence as degree of membership, prediction rejection as degree of non-membership, and ambiguity or model disagreement as degree of indeterminacy. The global parameter of the updated model affects the reliability of the attributes and the weighting in the decision-making process of INS-Sugeno-Weber, which allows the decision fusion to be adaptive and consistent throughout the entire drone swarm.

##  Aggregation operators of *INS* by using SW operator

This section continues to establish DM solutions to multi-criteria situations whereby complex autonomous pipeline monitoring through the application of complex AgOs is established to INS and SW norms. The proposed operators will focus on the inclusion of heterogeneous sensor data such as thermal imagery, gas concentration levels, and evolving acoustic signals, which are collected with federated swarms of Airbots through federated Edge-AI-enabled drones. Here, we say and justify the most important rubberizing aggregation operators, including the weighted SW neutrosophic averaging and geometric forms; both of these can be applied to the synthesis of uncertain, imprecise, and even conflicting information with success. With the help of the hybrid flexibility of the INS-SW combination, such operators increase the precision, stability, and reliability of the DM process in the presence of uncertainty. This methodology gives the possibility to interpret the field data stronger and offers a dynamic method of assessment of various factors of operations in real time. Therefore, it enhances greatly the accuracy and reliability of leak detection within natural gas pipeline networks even in the changing and difficult environmental conditions. This combination of the INS logic and the SW-based aggregation process is such that the indirect dependencies between criteria, e.g., sensor credibility, communication latency, and environmental, are systematically encapsulated and manifested in the end-result decision.

### Intuitionistic neutrosophic weighted arithmetic Sugeno-Weber operator $( INWA_{SW})$

Operators, including, $INWA_{SW}$ are a powerful MCDM tool, which is aimed at eliminating uncertainties and imprecision that happen in complex settings. This operator provides more refined and complete description of the information of the leak detection of the current time as compared to the traditional methods, namely autonomous drone swarms have already measured the truth, indeterminacy and falsity attributes of sensor data. Being a combination of the weighted arithmetic methodology and the flexible SW norm, the combination of the two allows utilizing the $INWA_{SW}$ to make nonlinear inferences between diverse criteria, e.g. thermal imaging, gas concentration readings, and acoustic signals. This technique provides a way to manage a large number of sources of uncertainty without simplification, which is particularly helpful in the situations when the data may be noisy, contradictory, or incomplete. As a result, it enables the detection decisions to be made more reliably, which reduces the safety and efficiency of the functioning of federated Edge-AI systems that are deployed to keep a track of natural gas pipelines.

#### Definition 4.1

Let $\alpha _{\iota \sigma }= \Big \{{\tau }_{\alpha _{\iota \sigma }},{\iota _{\alpha _{\iota \sigma }}},{\phi }_{\alpha _{\iota \sigma }}\Big \}$ and $i \in N$ (where N is set of natural numbers) be a selection of *INS*s and the $INWA_{SW}$ $INWA_{SW}: INS^{N}\rightarrow INS$ is defined as

$$\begin{aligned} INWA_{SW}(\alpha _{\sigma _1},\alpha _{\sigma _2},\alpha _{\sigma 3} ..., \alpha _{\sigma n})=\sum _{i=1}^{n}\omega _{i}\alpha _{\sigma _{i}} \end{aligned}$$

Where $0\le \omega _{i}\le 1, \sum _{1}^{n}\omega _{i}=1$ and $\omega _{i}$ signify weight vector (W-V).

#### Theorem 4.2

Let $\alpha _{\sigma _{i}}=\Big \{{\tau }_{\alpha _{\iota \sigma }},{\iota }_{\alpha _{\iota \sigma }},{\phi }_{\alpha _{\iota \sigma }}\Big \}$ be a selection of *INS*s, where $i\in N$ then $INWA_{SW}$ is defined as:

$$\begin{aligned} INWA_{SW}(\alpha _{\sigma _1},\alpha {\sigma _2},\alpha _{\sigma 3}..., \alpha _{\sigma n})= & \sum _{i=1}^{n}\omega _{i}\alpha _{\sigma _{i}}\\= & \left\{ \begin{array}{c} \Bigg ({\frac{1+\gimel }{\gimel }\Big (1-\prod _{i=1}^{n}(1-{\tau }_{\alpha _{\sigma }}(\frac{\gimel }{1+\gimel }))^{\omega _{i}}\Big )}, \\ {\Big ((1+\gimel )\prod _{i=1}^{n}\Big (\frac{\gimel {\iota }_{\alpha _{\sigma }}+1}{1+\gimel }\Big )^{\omega _{i}}-1\Big )\frac{1}{\gimel }}, \\ {\Big ((1+\gimel )\prod _{i=1}^{n}\Big (\frac{\gimel {\phi }_{\alpha _{\sigma }}+1}{1+\gimel }\Big )^{\omega _{i}}-1\Big )\frac{1}{\gimel }}\Bigg ) \end{array} \right\} \end{aligned}$$

#### Proof

If $n=2$, we will use the mathematical induction approach to prove it using the operations (1) and (3) of Definition 3.1.

$$\begin{aligned} \omega _{1}\odot \alpha _{\sigma _{1}}= & \left\{ \begin{array}{c} \Bigg ({\frac{1+\gimel }{\gimel }(1-(1-{\tau }_{\alpha _{\sigma 1}}(\frac{\gimel }{1+\gimel }))^{\omega _{1}})},\\ {((1+\gimel )(\frac{\gimel {\iota }_{\alpha _{\sigma 1}}+1}{1+\gimel })^{\omega _{1}}-1)\frac{1}{\gimel }},\\ {((1+\gimel )(\frac{\gimel {\phi }_{\alpha _{\sigma 1}}+1}{1+\gimel })^{\omega _{1}}-1)\frac{1}{\gimel }}\Bigg ) \end{array} \right\} .\\ \omega _{2}\odot \alpha _{\sigma _2}= & \left\{ \begin{array}{c} \Bigg ({\frac{1+\gimel }{\gimel }(1-(1-{\tau }_{\alpha _{\sigma 2}}(\frac{\gimel }{1+\gimel }))^{\omega _{2}})},\\ {((1+\gimel )(\frac{\gimel {\iota }_{\alpha _{\sigma 2}}+1}{1+\gimel })^{\omega _{2}}-1)\frac{1}{\gimel }},\\ {((1+\gimel )(\frac{\gimel {\phi }_{\alpha _{\sigma 2}}+1}{1+\gimel })^{\omega _{2}}-1)\frac{1}{\gimel }}\Bigg ) \end{array} \right\} . \end{aligned}$$

$$\begin{aligned}&INWA_{SW}(\alpha _{\sigma _1}\oplus \alpha _{\sigma _2})=\sum _{i=1}\omega _{i}\alpha _{\sigma _{i}} =\omega _{1}\alpha _{\sigma _1}\oplus \omega _{2}\alpha _{\sigma _2}\\&=\left\{ \begin{array}{c} \Bigg ({\frac{1+\gimel }{\gimel }(1-(1-{\tau }_{\alpha _{\sigma 1}}(\frac{\gimel }{1+\gimel }))^{\omega _{1}})},\\ {((1+\gimel )(\frac{\gimel {\iota }_{\alpha _{\sigma 1}}+1}{1+\gimel })^{\omega _{1}}-1)\frac{1}{\gimel }},\\ {((1+\gimel )(\frac{\gimel {\phi }_{\alpha _{\sigma 1}}+1}{1+\gimel })^{\omega _{1}}-1)\frac{1}{\gimel }}\Bigg ) \end{array} \right\} \bigoplus \left\{ \begin{array}{c} \Bigg ({\frac{1+\gimel }{\gimel }(1-(1-{\tau }_{\alpha _{\sigma 2}}(\frac{\gimel }{1+\gimel }))^{\omega _{2}})},\\ {((1+\gimel )(\frac{\gimel {\iota }_{\alpha _{\sigma 2}}+1}{1+\gimel })^{\omega _{2}}-1)\frac{1}{\gimel }},\\ {((1+\gimel )(\frac{\gimel {\phi }_{\alpha _{\sigma 2}}+1}{1+\gimel })^{\omega _{2}}-1)\frac{1}{\gimel }}\Bigg ) \end{array} \right\} .\\&=\left\{ \begin{array}{c} \Bigg (\Big (\Big (\frac{1+\gimel }{\gimel }\Big (1-\Big (1-\tau _{\alpha _{\sigma 1}}\Big (\frac{\gimel }{1+\gimel }\Big )\Big )^{ \omega _{1}}\Big )\Big )+ \Big (\frac{1+\gimel }{\gimel }\Big (1-\Big (1-\tau _{\alpha _{\sigma 2}}\Big (\frac{\gimel }{1+\gimel }\Big )\Big )^{ \omega _{2}}\Big )\Big )- \\ \frac{\gimel }{1+\gimel }\Big (\frac{1+\gimel }{\gimel }\Big (1-\Big (1-\tau _{\alpha _{\sigma 1}}\Big (\frac{\gimel }{1+\gimel }\Big )\Big )^{ \omega _{1}}\Big )\Big ) \Big (\frac{1+\gimel }{\gimel }\Big (1-\Big (1-\tau _{\alpha _{\sigma 2}} \Big (\frac{\gimel }{1+\gimel }\Big )\Big )^{ \omega _{2}}\Big )\Big )\Big ),\\ \Big (\Big ((\frac{1}{1+\gimel }\Big (\Big (((1+\gimel )\Big (\frac{\gimel {\iota }_{\alpha _{\sigma 1}}+1}{1+\gimel }\Big )^{\omega _{1}}-1\Big )\frac{1}{\gimel })+ \Big (\Big ((1+\gimel ) \Big (\frac{\gimel {\iota }_{\alpha _{\sigma 2}}+1}{1+\gimel }\Big )^{\omega _{2}}-1\Big )\frac{1}{\gimel }\Big )\\ -1+\gimel \Big (\Big ((1+\gimel )\Big (\frac{\gimel {\iota }_{\alpha {\sigma 1}}+1}{1+\gimel } \Big )^{\omega _{1}}-1\Big )\frac{1}{\gimel }) \Big (\Big ((1+\gimel )\Big (\frac{\gimel {\iota }_{\alpha {\sigma 2}}+1}{1+\gimel }\Big )^{\omega _{2}}-1\Big ) \frac{1}{\gimel }\Big )\Big ))\Big )\Big ) \\ \Big (\Big ((\frac{1}{1+\gimel }\Big (\Big (((1+\gimel )\Big (\frac{\gimel {\phi }_{\alpha _{\sigma 1}}+1}{1+\gimel }\Big )^{\omega _{1}}-1\Big )\frac{1}{\gimel }) + \Big (\Big ((1+\gimel )\Big (\frac{\gimel {\phi }_{\alpha _{\sigma 2}}+1}{1+\gimel }\Big )^{\omega _{2}}-1\Big )\frac{1}{\gimel }\Big )\\ -1+\gimel \Big (\Big ((1+\gimel )\Big (\frac{\gimel {\phi }_{\alpha _{\sigma 1}}+1}{1+\gimel }\Big )^{\omega _{1}}-1\Big )\frac{1}{\gimel }) \Big (\Big ((1+\gimel )\Big (\frac{\gimel {\phi }_{\alpha _{\sigma 2}}+1}{1+\gimel }\Big )^{\omega _{2}}-1\Big )\frac{1}{\gimel }\Big )\Big )) \Big )\Big )\Bigg ) \end{array} \right\} \end{aligned}$$

$$\begin{aligned}= & \left\{ \begin{array}{c} \Bigg ({\Big (\frac{1+\gimel }{\gimel }\Big (1-\Big (1-\tau _{\alpha _{\sigma 1}}\Big (\frac{\gimel }{1+\gimel }\Big )\Big )^{ \omega _{1}}\Big (1-\tau _{\alpha _{\sigma 1}}\Big (\frac{\gimel }{1+\gimel }\Big )\Big )^{ \omega _{2}}\Big )\Big )}\Bigg ) \\ {\frac{1}{\gimel }\Big (-1+(1+\gimel )\Big (\frac{\gimel \iota _{\alpha _{\sigma 1}}+1}{1+\gimel }\Big )^{\omega _{1}} \Big (\frac{\gimel \iota _{\alpha _{\sigma 2}}+1}{1+\gimel }\Big )^{\omega _{2}}\Big )} \\ {\frac{1}{\gimel }\Big (-1+(1+\gimel )\Big (\frac{\gimel \phi _{\alpha _{\sigma 1}}+1}{1+\gimel }\Big )^{\omega _{1}}\Big )} \Bigg ( \frac{\gimel , \phi _{\alpha _{\sigma 2}+1}}{1+\gimel } \Bigg )^{\omega _{2}} \end{array} \right\} . \end{aligned}$$

This implies that

$$\begin{aligned} INWA_{SW}(\alpha _{\sigma _1}\oplus \alpha _{\sigma _2})= & \left\{ \begin{array}{c} \Bigg ({\frac{1+\gimel }{\gimel }\Big (1-\prod _{i=1}^{n}(1-{\tau }_{\alpha _{\iota \sigma }}(\frac{\gimel }{1+\gimel }))^{\omega _{i}}\Big )}, \\ \Big ( (1+\gimel )\prod _{i=1}^{n} \left( \frac{\gimel \, \iota _{\alpha _{\iota \sigma }+1}}{1+\gimel } \right) ^{\omega _{i}} - 1 \Big )\frac{1}{\gimel }, \\ {\Big ((1+\gimel )\prod _{i=1}^{n}\Big (\frac{\gimel {\phi }_{\alpha _{\iota \sigma }}+1}{1+\gimel }\Big )^{\omega _{i}}-1\Big )\frac{1}{\gimel }}\Bigg ) \end{array} \right\} \end{aligned}$$

if n = b then:

$$\begin{aligned} INWA_{SW}(\alpha _{\sigma _1},\alpha _{\sigma _2},\alpha _{\sigma _3}..., \alpha _{\sigma _b})= & \sum _{i=1}^{b}\omega _{i}\alpha _{\sigma _{i}}\\= & \left\{ \begin{array}{c} \Bigg ({\frac{1+\gimel }{\gimel }\Big (1-\prod _{i=1}^{b}(1-{\tau }_{\alpha _{\iota \sigma }}(\frac{\gimel }{1+\gimel }))^{\omega _{i}}\Big )}, \\ \Big ((1+\gimel )\prod _{i=1}^{b}\Big (\frac{\gimel {\iota }_{\alpha _{\iota \sigma }}+1}{1+\gimel }\Big )^{\omega _{i}}-1\Big ){\frac{1}{\gimel }}, \\ \Big ((1+\gimel )\prod _{i=1}^{b}\Big (\frac{\gimel {\phi }_{\alpha _{\iota \sigma }}+1}{1+\gimel }\Big )^{\omega _{i}}-1\Big ){\frac{1}{\gimel }}\Bigg ) \end{array} \right\} \end{aligned}$$

if n = b+1 then:

$$\begin{aligned} INWA_{SW}(\alpha _{\sigma _1},\alpha _{\sigma _2},\alpha _{\sigma _3}..., \alpha _{\sigma _ {b+1}})= & \sum _{i=1}^{b}\omega _{i}\alpha _{\iota \sigma }\oplus \omega _{b+1}\alpha _{\sigma _{b+1}}\\= & \left\{ \begin{array}{c} \Bigg ({\frac{1+\gimel }{\gimel }\Big (1-\prod _{i=1}^{b}(1-{\tau }_{\alpha _{\iota \sigma }}(\frac{\gimel }{1+\gimel }))^{\omega _{i}}\Big )}, \\ {\Big ((1+\gimel )\prod _{i=1}^{b}\Big (\frac{\gimel {\iota }_{\alpha _{\iota \sigma }}+1}{1+\gimel }\Big )^{\omega _{i}}-1\Big )\frac{1}{\gimel }}, \\ {\Big ((1+\gimel )\prod _{i=1}^{b}\Big (\frac{\gimel {\phi }_{\alpha _{\iota \sigma }}+1}{1+\gimel }\Big )^{\omega _{i}}-1\Big )\frac{1}{\gimel }}\Bigg ) \end{array} \right\} \oplus \omega _{b+1}\alpha _{\sigma _{b+1}}\\= & \left\{ \begin{array}{c} \Bigg ({\frac{1+\gimel }{\gimel }\Big (1-\prod _{i=1}^{b+1}(1-{\tau }_{\alpha _{\iota \sigma }}(\frac{\gimel }{1+\gimel }))^{\omega _{i}}\Big )}, \\ {\Big ((1+\gimel )\prod _{i=1}^{b+1}\Big (\frac{\gimel {\iota }_{\alpha _{\iota \sigma }}+1}{1+\gimel }\Big )^{\omega _{i}}-1\Big )\frac{1}{\gimel }}, \\ {\Big ((1+\gimel )\prod _{i=1}^{b+1}\Big (\frac{\gimel {\phi }_{\alpha _{\iota \sigma }}+1}{1+\gimel }\Big )^{\omega _{i}}-1\Big )\frac{1}{\gimel }}\Bigg ) \end{array} \right\} \end{aligned}$$

This implies that b+1 holds. Hence it is true for all n, and it completes the proof. $\square$

#### Property 4.3

**Idempotency: **Let $\alpha _{\sigma _i}= \Big \{{\tau }_{i},{\iota }_{i},{\phi }_{i}\Big \}$ and $i \in N$ be a selection of *INS*s if all the $\alpha _{\sigma _i}$ are identical, then

$$\begin{aligned} INWA_{SW}(\alpha _{\sigma _1},\alpha _{\sigma _2},\alpha _{\sigma _3} ..., \alpha _{\sigma _n})=\alpha _{\sigma } \end{aligned}$$

#### Proof

let all the $\alpha _{\sigma _i}$ be identical, and we know that

$$\begin{aligned} INWA_{SW}(\alpha _{\sigma _1},\alpha _{\sigma _2},\alpha _{\sigma _3}..., \alpha _{\sigma _n})= & \sum _{i=1}^{n}\omega _{i}\alpha _{\sigma _{i}}\\= & \left\{ \begin{array}{c} \Bigg ({\frac{1+\gimel }{\gimel }\Big (1-\prod _{i=1}^{n}(1-{\tau }_{\alpha _{\iota \sigma }}(\frac{\gimel }{1+\gimel }))^{\omega _{i}}\Big )}, \\ {\Big ((1+\gimel )\prod _{i=1}^{n}\Big (\frac{\gimel {\iota }_{\alpha _{\iota \sigma }}+1}{1+\gimel }\Big )^{\omega _{i}}-1\Big )\frac{1}{\gimel }}, \\ {\Big ((1+\gimel )\prod _{i=1}^{n}\Big (\frac{\gimel {\phi }_{\alpha _{\iota \sigma }}+1}{1+\gimel }\Big )^{\omega _{i}}-1\Big )\frac{1}{\gimel }}\Bigg ) \end{array} \right\} \\= & \left\{ \begin{array}{c} \Bigg ({\frac{1+\gimel }{\gimel }\Big (1-(1-{\tau }_{\alpha _{\iota \sigma }}(\frac{\gimel }{1+\gimel }))^{\sum _{i=1}^{n}\omega _{i}}\Big )}, \\ {\Big ((1+\gimel )\Big (\frac{\gimel {\iota }_{\alpha _{\iota \sigma }}+1}{1+\gimel }\Big )^{\sum _{i=1}^{n}\omega _{i}}-1\Big )\frac{1}{\gimel }}, \\ {\Big ((1+\gimel )\Big (\frac{\gimel {\phi }_{\alpha _{\iota \sigma }}+1}{1+\gimel }\Big )^{\sum _{i=1}^{n}\omega _{i}}-1\Big )\frac{1}{\gimel }}\Bigg ) \end{array} \right\} \\= & \left\{ \begin{array}{c} \Bigg ({\frac{1+\gimel }{\gimel }\Big (1-(1-{\tau }_{\alpha _{\iota \sigma }}(\frac{\gimel }{1+\gimel }))\Big )}, \\ {\Big ((1+\gimel )\Big (\frac{\gimel {\iota }_{\alpha _{\iota \sigma }}+1}{1+\gimel }\Big )-1\Big )\frac{1}{\gimel }}, \\ {\Big ((1+\gimel )\Big (\frac{\gimel {\phi }_{\alpha _{\iota \sigma }}+1}{1+\gimel }\Big )-1\Big )\frac{1}{\gimel }}\Bigg ) \end{array} \right\} \\= & \alpha _{\sigma }. \end{aligned}$$

$$\square$$

#### Property 4.4

**Monotonicity:** Let $\alpha _{\sigma _i}= \Big \{{\tau }_{\alpha _{\sigma _i}},{\iota }_{\alpha _{\sigma _i}},{\phi }_{\alpha _{\sigma _i}}\Big \}$ and $\alpha _{\sigma _{i'}}= \Big \{{\tau }_{\sigma _i'},{\iota }_{\sigma _i'},{\phi }_{\sigma _i'}\Big \}$ $i, i'={1,2,3...,n}$ be a assemblages of *INS* so as $\alpha _{\sigma _i}\subseteq \alpha _{\sigma _i'}$ then,

$$\begin{aligned} INWA_{SW}(\alpha _{\sigma _1},\alpha _{\sigma _2},\alpha _{\sigma _3} ..., \alpha _{\sigma _n}) \le INWA_{SW}(\alpha _{\sigma _1'},\alpha _{\sigma _2'},\alpha _{\sigma _3'} ..., \alpha _{\sigma _n'}) \end{aligned}$$

#### Proof

As $\alpha _{\sigma _i}\subseteq \alpha _{\sigma _i'}$ Thus ${\tau }_{\alpha _{\sigma _i}}\le {\tau }_{\alpha _{\sigma _i'}}$ , ${\iota }_{\alpha _{\sigma _i}}\le {\iota }_{\alpha _{\sigma _i'}}$ ${\phi }_{\alpha _{\sigma _i}}\ge {\phi }_{\alpha _{\sigma _i'}}$ this implies that

$$\begin{aligned} {\frac{1+\gimel }{\gimel }(1-(1-\prod _{i=1}^{n}{\tau }_{\alpha _{\sigma _i}}(\frac{\gimel }{1+\gimel }))^{\omega _{i}})}\le & {\frac{1+\gimel }{\gimel }(1-(1-\prod _{i'=1}^{n}{\tau }_{\alpha _{\sigma _i'}}(\frac{\gimel }{1+\gimel }))^{\omega _{i'}})}),\\ {((1+\gimel )(\frac{\gimel \prod _{i=1}^{n}{\iota }_{\alpha _{\sigma _i}}+1}{1+\gimel })^{\omega _{i}}-1)\frac{1}{\gimel }}\le & {((1+\gimel )(\frac{\gimel \prod _{i'=1}^{n}{\iota }_{\alpha _{\sigma _i'}}+1}{1+\gimel })^{\omega _{i'}}-1)\frac{1}{\gimel }},\\ {((1+\gimel )(\frac{\gimel \prod _{i=1}^{n}{\phi }_{\alpha _{\sigma _i}}+1}{1+\gimel })^{\omega _{i}}-1)\frac{1}{\gimel }}\ge & {((1+\gimel )(\frac{\gimel \prod _{i'=1}^{n}{\phi }_{\alpha _{\sigma _i'}}+1}{1+\gimel })^{\omega _{i'}}-1)\frac{1}{\gimel }}, \end{aligned}$$

this implies that

$$\begin{aligned} \left\{ \begin{array}{c} {\frac{1+\gimel }{\gimel }\Big (1-\prod _{i=1}^{n}(1-{\tau }_{\alpha _{\sigma _i}}(\frac{\gimel }{1+\gimel }))^{\omega _{i}}\Big )}, \\ {\Big ((1+\gimel )\prod _{i=1}^{n}\Big (\frac{\gimel {\iota }_{\alpha _{\sigma _i}}+1}{1+\gimel }\Big )^{\omega _{i}}-1\Big )\frac{1}{\gimel }}, \\ {\Big ((1+\gimel )\prod _{i=1}^{n}\Big (\frac{\gimel {\phi }_{\alpha _{\sigma _i}}+1}{1+\gimel }\Big )^{\omega _{i}}-1\Big )\frac{1}{\gimel }}\Big ) \end{array} \right\} \le \left\{ \begin{array}{c} {\frac{1+\gimel }{\gimel }\Big (1-\prod _{i'=1}^{n}(1-{\tau }_{\alpha _{\sigma _i'}}(\frac{\gimel }{1+\gimel }))^{\omega _{i'}}\Big )}, \\ {\Big ((1+\gimel )\prod _{i'=1}^{n}\Big (\frac{\gimel {\iota }_{\alpha _{\sigma _i'}}+1}{1+\gimel }\Big )^{\omega _{i'}}-1\Big )\frac{1}{\gimel }}, \\ {\Big ((1+\gimel )\prod _{i'=1}^{n}\Big (\frac{\gimel {\phi }_{\alpha _{\sigma _i'}}+1}{1+\gimel }\Big )^{\omega _{i'}}-1\Big )\frac{1}{\gimel }}\Big ) \end{array} \right\} \end{aligned}$$

Hence

$$\begin{aligned} INWA_{SW}(\alpha _{\sigma _1},\alpha _{\sigma _2},\alpha _{\sigma _3} ... \alpha _{\sigma _n}) \le INWA_{SW}(\alpha _{\sigma _1'},\alpha _{\sigma _2'},\alpha _{\sigma _3'} ..., \alpha _{\sigma _n'}) \end{aligned}$$

$$\square$$

#### Property 4.5

**Boundedness:** Let $\alpha _{\sigma _i}= \Big \{{\tau }_{\alpha _{\sigma _i}},{\iota }_{\alpha _{\sigma _i}},{\phi }_{\alpha _{\sigma _i}}\Big \}$ and $i \in N$ be a assemblages of *INS* so as $\alpha _{\sigma _A}=\max \limits _{i}^{}\alpha _{\sigma _i}$ and $\alpha _{\sigma _B}=\min \limits _{i}^{}$ $\alpha _{\sigma _i}$ then,

$$\begin{aligned} \alpha _{\sigma _B}\le INWA_{SW}(\alpha _{\sigma _1},\alpha _{\sigma _2},\alpha _{\sigma _3} ..., \alpha _{\sigma _n})\le \alpha _{\sigma _A} \end{aligned}$$

#### Proof

The proof is straightforward. $\square$

#### Definition 4.6

Let $\alpha _{\sigma _i}= \Big \{{\tau }_{\alpha _{\sigma _i}},{\iota }_{\alpha _{\sigma _i}},{\phi }_{\alpha _{\sigma _i}}\Big \}$ and $i \in N$ be a selection of *INS*s and the $INOWA_{SW} INOWA_{SW}: INS^{N}\rightarrow INS$ is defined as

$$\begin{aligned} INOWA_{SW}(\alpha _{\sigma _1},\alpha _{\sigma _2},\alpha _{\sigma _3} ..., \alpha _{\sigma _n})=\sum _{i=1}^{n}\omega _{i}\alpha _{\sigma _{\delta (i)}} \end{aligned}$$

with n dimensions so as ith highest weighted value (HWV) is, $\alpha _{\sigma _i}$ as a result, by the overall order $\alpha _{\sigma _1}\ge \alpha _{\sigma _2} \ge \alpha _{\sigma _3} \ge ...\ge \alpha _{\sigma _n}$ $0\le \omega _{i}\le 1, \sum _{1}^{n}\omega _{i}=1$ and $\omega _{i}$ signifies W-V.

#### Theorem 4.7

Let $\alpha _{\sigma _{i}}=\Big \{{\tau }_{\alpha _{\sigma _i}},{\iota }_{\alpha _{\sigma _i}},{\phi }_{\alpha _{\sigma _i}}\Big \}$ be a selection of *INS*s, where $i\in N$ then $INOWA_{SW}$ is defined as:

$$\begin{aligned} INOWA_{SW}(\alpha _{\sigma _1},\alpha _{\sigma _2},\alpha _{\sigma _3}... \alpha _{\sigma _n})= & \sum _{i=1}^{n}\omega _{i}\alpha _{\sigma _{\delta (i)}}\\= & \left\{ \begin{array}{c} \Bigg ({\frac{1+\gimel }{\gimel }\Big (1-\prod _{i=1}^{n}(1-{\tau }_{\delta (i)}(\frac{\gimel }{1+\gimel }))^{\omega _{i}}\Big )}, \\ {\Big ((1+\gimel )\prod _{i=1}^{n}\Big (\frac{\gimel {\iota }_{\delta (i)}+1}{1+\gimel }\Big )^{\omega _{i}}-1\Big )\frac{1}{\gimel }}, \\ {\Big ((1+\gimel )\prod _{i=1}^{n}\Big (\frac{\gimel {\phi }_{\delta (i)}+1}{1+\gimel }\Big )^{\omega _{i}}-1\Big )\frac{1}{\gimel }}\Bigg ) \end{array} \right\} \end{aligned}$$

with n dimensions, so as $\alpha _{\sigma _i}$ is the ith HWV; consequently, the total order $\alpha _{\sigma _1}\ge \alpha _{\sigma _2} \ge \alpha _{\sigma _3} \ge ...\ge \alpha _{\sigma _n}$ and $0\le \omega _{i}\le 1, \sum _{1}^{n}\omega _{i}=1$ and $\omega _{i}$ signify W-V.

#### Proof

The proof relates to the $INWA_{SW}$ operator above. Hence, we avoid it. $\square$

#### Property 4.8

**Idempotency:** Let $\alpha _{\sigma _i}= \Big \{{\tau }_{\alpha _{\sigma _i}},{\iota }_{\alpha _{\sigma _i}},{\phi }_{\alpha _{\sigma _i}}\Big \}$ and $i \in N$ be a selection of *INS*s if all them $\alpha _{\sigma _i}$ are identical, then

$$\begin{aligned} INOWA_{SW}(\alpha _{\sigma _1},\alpha _{\sigma _2},\alpha _{\sigma _3} ..., \alpha _{\sigma _n})=\alpha _{\sigma } \end{aligned}$$

#### Property 4.9

**Monotonicity:** Let $\alpha _{\sigma _i}= \Big \{{\tau }_{\alpha _{\sigma _i}},{\iota }_{\alpha _{\sigma _i}},{\phi }_{\alpha _{\sigma _i}}\Big \}$ and $\alpha _{\sigma _{i'}}= \Big \{{\tau }_{\alpha _{\sigma _i'}},{\iota }_{\alpha _{\sigma _i'}},{\phi }_{\alpha _{\sigma _i'}}\Big \}$ where $i, i'={1,2,3...,n}$ be a assemblages of *INS* so that $\alpha _{\sigma _i}\subseteq \alpha _{\sigma _i'}$ then,

$$\begin{aligned} INOWA_{SW}(\alpha _{\sigma _1},\alpha _{\sigma _2},\alpha _{\sigma _3} ..., \alpha _{\sigma _n}) \le INOWA_{SW}(\alpha _{\sigma _1'},\alpha _{\sigma _2'},\alpha _{\sigma _3'} ..., \alpha _{\sigma _n'}) \end{aligned}$$

#### Property 4.10

**Boundedness:** Let $\alpha _{\sigma _i}= \Big \{{\tau }_{\alpha _{\sigma _i}},{\iota _{\sigma _i}},{\phi }_{\alpha _{\sigma _i}}\Big \}$ and $i \in N$ be a assemblages of *INS* so as $\alpha _{\sigma _A}=$ max $\alpha _{\sigma _i}$ and $\alpha _{\sigma _B}= \min \alpha _{\sigma _i}$ then,

$$\begin{aligned} \alpha _{\sigma _B}\le INOWA_{SW}(\alpha _{\sigma _1},\alpha _{\sigma _2},\alpha _{\sigma _3} ..., \alpha _{\sigma _n})\le \alpha _{\sigma _A} \end{aligned}$$

#### Definition 4.11

Let $\alpha _{\sigma _i}= \Big \{{\tau }_{\alpha _{\sigma _i}},{\iota }_{\alpha _{\sigma _i}},{\phi }_{\alpha _{\sigma _i}}\Big \}$ and $i \in N$ be a selection of *INS*s and the $INHWA_{SW}$ so as $INHWA_{SW}: INS^{N}\rightarrow INS$ is defined as

$$\begin{aligned} INHWA_{SW}(\alpha _{\sigma _1},\alpha _{\sigma _2},\alpha _{\sigma _3} ..., \alpha _{\sigma _n})=\sum _{i=1}^{n}\omega _{i}\alpha '_{\sigma _{\delta (i)}} \end{aligned}$$

with n dimensions so as ith HWV is $\alpha _{\sigma _{\delta (i)}}$ and $\alpha '_{\sigma _i}=n{\lambda _{i}}\alpha _{\sigma _i},(i\in N)$ , $0\le \lambda _{i}\le 1, \sum _{1}^{n}\lambda _{i}=1$ and $\lambda _{i}$ signify W-V also ${\lambda _{i}}$ is the associated W-V $0\le \lambda _{i}\le 1, \sum _{1}^{n}\lambda _{i}=1$ and the balancing coefficient (B-C) is n.

#### Theorem 4.12

Let $\alpha _{\sigma _{i}}=\Big \{{\tau }_{\alpha _{\sigma _i}},{\iota }_{\alpha _{\sigma _i}},{\phi }_{\alpha _{\sigma _i}}\Big \}$ be a selection of *INS*s, where $i\in N$ then $INHWA_{SW}$ is defined as:

$$\begin{aligned} INHWA_{SW}(\alpha _{\sigma _1},\alpha _{\sigma _2},\alpha _{\sigma _3}..., \alpha _{\sigma _n})= & \sum _{i=1}^{n}{\lambda _{i}}\alpha '_{\sigma _{\delta (i)}}\\= & \left\{ \begin{array}{c} \Bigg ({\frac{1+\gimel }{\gimel }\Big (1-\prod _{i=1}^{n}(1-{\tau '}_{\delta (i)}(\frac{\gimel }{1+\gimel }))^{\lambda _{i}}\Big )}, \\ {\Big ((1+\gimel )\prod _{i=1}^{n}\Big (\frac{\gimel {\iota '}_{\delta (i)}+1}{1+\gimel }\Big )^{\lambda _{i}}-1\Big )\frac{1}{\gimel }}, \\ {\Big ((1+\gimel )\prod _{i=1}^{n}\Big (\frac{\gimel {\phi '}_{\delta (i)}+1}{1+\gimel }\Big )^{\lambda _{i}}-1\Big )\frac{1}{\gimel }}\Bigg ) \end{array} \right\} \end{aligned}$$

with n dimensions so as ith HWV is $\alpha _{\sigma _{\delta (i)}}$ and $\alpha '_{\sigma _i}=n{\lambda _{i}}\alpha _{\sigma _i},(i\in N)$ , $0\le \omega _{i}\le 1, \sum _{1}^{n}\omega _{i}=1$ and $\omega _{i}$ signify W-V also ${\lambda _{i}}$ is the associated W-V $0\le \lambda _{i}\le 1, \sum _{1}^{n}\lambda _{i}=1$ and the B-C is n.

#### Property 4.13

**Idempotency :**Let $\alpha _{\sigma _i}= \Big \{{\tau }_{\alpha _{\sigma _i}},{\iota }_{\alpha _{\sigma _i}},{\phi }_{\alpha _{\sigma _i}}\Big \}$ and $i \in N$ be a selection of *INS*s. If all them $\alpha _{\sigma _i}$ are identical then

$$\begin{aligned} INHWA_{SW}(\alpha _{\sigma _1},\alpha _{\sigma _2},\alpha _{\sigma _3} ..., \alpha _{\sigma _n})=\alpha _{\sigma } \end{aligned}$$

#### Property 4.14

**Monotonicity:** Let $\alpha _{\sigma _i}= \Big \{{\tau }_{\alpha _{\sigma _i}},{\iota }_{\alpha _{\sigma _i}},{\phi }_{\alpha _{\sigma _i}}\Big \}$ and $\alpha _{\sigma _{i'}}= \Big \{{\tau }_{\alpha _{\sigma _i'}},{\iota }_{\alpha _{\sigma _i'}},{\phi }_{\alpha _{\sigma _i'}}\Big \}$ where $i, i'={1,2,3...,n}$ be a assemblages of *INS* so as $\alpha _{\sigma _i}\subseteq \alpha _{\sigma _i'}$ then,

$$\begin{aligned} INHWA_{SW}(\alpha _{\sigma _1},\alpha _{\sigma _2},\alpha _{\sigma _3} ..., \alpha _{\sigma _n}) \le INHWA_{SW}(\alpha _{\sigma _1'},\alpha _{\sigma _2'},\alpha _{\sigma _3'} ..., \alpha _{\sigma _n'}) \end{aligned}$$

#### Property 4.15

**Boundedness:** Let $\alpha _{\sigma _i}= \Big \{{\tau }_{\alpha _{\sigma _i}},{\iota }_{\alpha _{\sigma _i}},{\phi }_{\alpha _{\sigma _i}}\Big \}$ and $i \in N$ be a assemblages of *INS* so as $\alpha _{\sigma _A}=$ max $\alpha _{\sigma _i}$ and $\alpha _{\sigma _B}=$ min $\alpha _{\sigma _i}$ then,

$$\begin{aligned} \alpha _{\sigma _B}\le INHWA_{SW}(\alpha _{\sigma _1},\alpha _{\sigma _2},\alpha _{\sigma _3} ..., \alpha _{\sigma _n})\le \alpha _{\sigma A} \end{aligned}$$

### Intuitionistic neutrosophic weighted geometric Sugeno-Weber operator $( INWG_{SW})$

In this subsection, a new operator is proposed, namely, a new $INWG_{SW}$ operator that can be applied to increase decision aggregation in uncertain environments. This operator is valuable since it will reflect the interactions between several decision criteria both synergistic and antagonistic, by incorporating the weighted geometric mean into a dynamic model. With this structure the weights of importance of the various criterion adaptively change in relation to the rest and guarantee balanced and context sensitive assessment. The added SW norm also enhances the soundness of the process of decision-making at the time of uncertainty and conflicting conditions. It is especially useful in complicated processes like real-time leak detection by federated Edge-AI autonomous drone swarm in natural gas pipeline oversight processes where sensor information can be unclear, inconsistent, or missing.

#### Definition 4.16

Let $\alpha _{\sigma _i}= \Big \{{\tau }_{\alpha _{\sigma _i}},{\iota }_{\alpha _{\sigma _i}},{\phi }_{\alpha _{\sigma _i}}\Big \}$ and $i \in N$ be a selection of *INS*s and the $INWG_{SW}$ $INWG_{SW}: INS^{N}\rightarrow INS$ is defined as

$$\begin{aligned} INWG_{SW}(\alpha _{\sigma _1},\alpha _{\sigma _2},\alpha _{\sigma _3} ..., \alpha _{\sigma _n})=\prod _{i=1}^{n}\alpha {^{\omega _{i}}_{\sigma _{i}}} \end{aligned}$$

Where $0\le \omega _{i}\le 1, \sum _{1}^{n}\omega _{i}=1$ and $\omega _{i}$ signify W-V.

#### Theorem 4.17

Let $\alpha _{\sigma _{i}}=\Big \{{\tau }_{\alpha _{\sigma _i}},{\iota }_{\alpha _{\sigma _i}},{\phi }_{\alpha _{\sigma _i}}\Big \}$ be a selection of *INS*s, where $i\in N$ then $INWG_{SW}$ is defined as:

$$\begin{aligned} INWG_{SW}(\alpha _{\sigma _1},\alpha _{\sigma _2},\alpha _{\sigma _3}..., \alpha _{\sigma _n})= & \prod _{i=1}^{n}\alpha _{\sigma _{i}}^{\omega _{i}}\\= & \left\{ \begin{array}{c} \Bigg ({\Big ((1+\gimel )\prod _{i=1}^{n}\Big (\frac{\gimel {\tau }_{\alpha _{\sigma _i}}+1}{1+\gimel }\Big )^{\omega _{i}}-1\Big )\frac{1}{\gimel }}\\ {\frac{1+\gimel }{\gimel }\Big (1-\prod _{i=1}^{n}\Big (1-{\iota }_{\alpha _{\sigma _i}}(\frac{\gimel }{1+\gimel })\Big )^{\omega _{i}}\Big )},\\ {\frac{1+\gimel }{\gimel }\Big (1-\prod _{i=1}^{n}\Big (1-{\phi }_{\alpha _{\sigma _i}}(\frac{\gimel }{1+\gimel })\Big )^{\omega _{i}}\Big )}\Bigg ), \end{array} \right\} \end{aligned}$$

with n dimensions so as ith HWV is $\alpha _{\sigma _{\Delta (i)}}$ and $\alpha '_{\sigma _i}=n{\lambda _{i}}\alpha _{\sigma _i}(i\in N$ , $0\le \omega _{i}\le 1, \sum _{1}^{n}\omega _{i}=1$ and $\omega _{i}$ signify W-V also ${\lambda _{i}}$ is the associated W-V $0\le \lambda _{i}\le 1, \sum _{1}^{n}\lambda _{i}=1$ and the B-C is n

#### Proof

If $n=2$, we will use the mathematical induction approach to prove it using the operations (1) and (3) of definition 3.1.

$$\begin{aligned} \alpha _{\sigma _1}^{\omega _{1}}= & \left\{ \begin{array}{c} \Bigg ({((1+\gimel )(\frac{\gimel {\tau }_{1}+1}{1+\gimel })^{\omega _{1}}-1)\frac{1}{\gimel }},\\ {\frac{1+\gimel }{\gimel }(1-(1-{\iota }_{1}(\frac{\gimel }{1+\gimel }))^{\omega _{1}})},\\ {\frac{1+\gimel }{\gimel }(1-(1-{\phi }_{1}(\frac{\gimel }{1+\gimel }))^{\omega _{1}})}\Bigg ) \end{array} \right\} .\\ \alpha _{\sigma _2}^{\omega _{2}}= & \left\{ \begin{array}{c} \Bigg ({((1+\gimel )(\frac{\gimel {\tau }_{1}+1}{1+\gimel })^{\omega _{2}}-1)\frac{1}{\gimel }},\\ {\frac{1+\gimel }{\gimel }(1-(1-{\iota }_{1}(\frac{\gimel }{1+\gimel }))^{\omega _{2}})},\\ {\frac{1+\gimel }{\gimel }(1-(1-{\phi }_{1}(\frac{\gimel }{1+\gimel }))^{\omega _{2}})}\Bigg ) \end{array} \right\} . \end{aligned}$$

$$\begin{aligned} & INWG_{SW}(\alpha _{\sigma _1}\otimes \alpha _{\sigma _2}) =\sum _{i=1}\alpha _{\sigma _{\alpha _{\sigma _i}}}^{\omega _{i}}=\alpha _{\sigma _1}^{\omega _{1}}\otimes \alpha _{\sigma _2}^{\omega _{2}}\\= & \left\{ \begin{array}{c} \Bigg ({((1+\gimel )(\frac{\gimel {\tau }_{1}+1}{1+\gimel })^{\omega _{1}}-1)\frac{1}{\gimel }},\\ {\frac{1+\gimel }{\gimel }(1-(1-{\iota }_{\alpha _{\sigma _i}}(\frac{\gimel }{1+\gimel }))^{\omega _{1}})},\\ {\frac{1+\gimel }{\gimel }(1-(1-{\phi }_{\alpha _{\sigma _i}}(\frac{\gimel }{1+\gimel }))^{\omega _{1}})}\Bigg )\\ \end{array} \right\} \bigotimes \left\{ \begin{array}{c} \Bigg ({((1+\gimel )(\frac{\gimel {\tau }_{1}+1}{1+\gimel })^{\omega _{2}}-1)\frac{1}{\gimel }},\\ {\frac{1+\gimel }{\gimel }(1-(1-{\iota }_{1}(\frac{\gimel }{1+\gimel }))^{\omega _{2}})},\\ {\frac{1+\gimel }{\gimel }(1-(1-{\phi }_{1}(\frac{\gimel }{1+\gimel }))^{\omega _{2}})}\Bigg ) \end{array} \right\} .\\ \end{aligned}$$

$$\begin{aligned}= & \left\{ \begin{array}{c} \Bigg (\Big ((\frac{1}{1+\gimel }\Big (\Big (((1+\gimel )\Big (\frac{\gimel {\tau }_{1}+1}{1+\gimel }\Big )^{\omega _{1}} -1\Big )\frac{1}{\gimel })+\Big (\Big ((1+\gimel )\Big (\frac{\gimel {\tau }_{1}+1}{1+\gimel }\Big )^{\omega _{2}}-1\Big )\frac{1}{\gimel }\Big )\\ -1+\gimel \Big (\Big ((1+\gimel )\Big (\frac{\gimel {\tau }_{1}+1}{1+\gimel }\Big )^{\omega _{1}}-1\Big ) \frac{1}{\gimel })\Big (\Big ((1+\gimel )\Big (\frac{\gimel {\tau }_{1}+1}{1+\gimel }\Big )^{\omega _{2}}-1\Big ) \frac{1}{\gimel }\Big )\Big )), \\ \Big (\Big (\Big (\frac{1+\gimel }{\gimel }\Big (1-\Big (1-\iota _{1}\Big (\frac{\gimel }{1+\gimel }\Big )\Big )^{ \omega _{1}}\Big )\Big )+\Big (\frac{1+\gimel }{\gimel }\Big (1-\Big (1-\iota _{1}\Big (\frac{\gimel }{1+\gimel }\Big )\Big )^{ \omega _{2}}\Big )\Big )- \\ \frac{\gimel }{1+\gimel }\Big (\frac{1+\gimel }{\gimel }\Big (1-\Big (1-\iota _{1}\Big (\frac{\gimel }{1+\gimel }\Big )\Big )^{ \omega _{1}}\Big )\Big )\Big (\frac{1+\gimel }{\gimel }\Big (1-\Big (1-\iota _{1}\Big (\frac{\gimel }{1+\gimel }\Big )\Big )^{ \omega _{2}}\Big )\Big ), \\ \Big (\Big (\Big (\frac{1+\gimel }{\gimel }\Big (1-\Big (1-\phi _{1}\Big (\frac{\gimel }{1+\gimel }\Big )\Big )^{ \omega _{1}}\Big )\Big )+\Big (\frac{1+\gimel }{\gimel }\Big (1-\Big (1-\phi _{1}\Big (\frac{\gimel }{1+\gimel }\Big )\Big )^{ \omega _{2}}\Big )\Big )- \\ \frac{\gimel }{1+\gimel }\Big (\frac{1+\gimel }{\gimel }\Big (1-\Big (1-\phi _{1}\Big (\frac{\gimel }{1+\gimel }\Big )\Big )^{ \omega _{1}}\Big )\Big )\Big (\frac{1+\gimel }{\gimel }\Big (1-\Big (1-\phi _{1}\Big (\frac{\gimel }{1+\gimel }\Big )\Big )^{ \omega _{2}}\Big )\Big )\Big )\Big )\Big )\Big )\Bigg ) \end{array} \right\} .\\= & \left\{ \begin{array}{c} \Bigg ({\frac{1}{\gimel }\Big (-1+(1+\gimel )\Big (\frac{\gimel \tau _{1}+1}{1+\gimel }\Big )^{\omega _{1}} \Big (\frac{\gimel \tau _{1}+1}{1+\gimel }\Big )^{\omega _{2}}\Big )} \\ {\Big (\frac{1+\gimel }{\gimel }\Big (1-\Big (1-\iota _{1}\Big (\frac{\gimel }{1+\gimel }\Big )\Big )^{ \omega _{1}}\Big (1-\iota _{1}\Big (\frac{\gimel }{1+\gimel }\Big )\Big )^{ \omega _{2}}\Big )} \\ {\Big (\frac{1+\gimel }{\gimel }\Big (1-\Big (1-\phi _{1}\Big (\frac{\gimel }{1+\gimel }\Big )\Big )^{ \omega _{1}}\Big (1-\phi _{1}\Big (\frac{\gimel }{1+\gimel }\Big )\Big )^{ \omega _{2}}\Big )\Big )}\Bigg ) \end{array} \right\} . \end{aligned}$$

this implies that

$$\begin{aligned} INWG{SW}(\alpha _{\sigma _1}\otimes \alpha _{\sigma _2})= & \left\{ \begin{array}{c} {\Big ((1+\gimel )\prod _{i=1}^{n}\Big (\frac{\gimel {\tau }_{i}+1}{1+\gimel }\Big )^{\omega _{i}}-1\Big )\frac{1}{\gimel }}\\ {\frac{1+\gimel }{\gimel }\Big (1-\prod _{i=1}^{n}\Big (1-{\iota }_{i}(\frac{\gimel }{1+\gimel })\Big )^{\omega _{i}}\Big )}\\ {\frac{1+\gimel }{\gimel }\Big (1-\prod _{i=1}^{n}\Big (1-{\phi }_{i}(\frac{\gimel }{1+\gimel })\Big )^{\omega _{i}}\Big )}, \end{array} \right\} \end{aligned}$$

if n = N then:

$$\begin{aligned} INWG_{SW}(\alpha _{\sigma _1},\alpha _{\sigma _2},\alpha _{\sigma _3}..., \alpha _{\sigma _N})= & \sum _{i=1}^{N}\alpha _{\sigma _{i}}^{\omega _{i}}\\= & \left\{ \begin{array}{c} \Bigg ({\Big ((1+\gimel )\prod _{i=1}^{N}\Big (\frac{\gimel {\tau }_{i}+1}{1+\gimel }\Big )^{\omega _{i}}-1\Big )\frac{1}{\gimel }}\\ \\ {\frac{1+\gimel }{\gimel }\Big (1-\prod _{i=1}^{N}\Big (1-{\iota }_{i}(\frac{\gimel }{1+\gimel })\Big )^{\omega _{i}}\Big )}\\ \\ {\frac{1+\gimel }{\gimel }\Big (1-\prod _{i=1}^{N}\Big (1-{\phi }_{i}(\frac{\gimel }{1+\gimel })\Big )^{\omega _{i}}\Big )}\Bigg ), \end{array} \right\} \end{aligned}$$

if n = N+1 then:

$$\begin{aligned} INWG_{SW}(\alpha _{\sigma _1},\alpha _{\sigma _2},\alpha _{\sigma _3}..., \alpha _{\sigma _{N+1}})= & \sum _{i=1}^{N}\alpha _{\sigma _{i}}^{\omega _{i}}\otimes \alpha _{\sigma _{N+1}}^{\omega _{N+1}}\\= & \left\{ \begin{array}{c} \Bigg ({\Big ((1+\gimel )\prod _{i=1}^{N}\Big (\frac{\gimel {\tau }_{i}+1}{1+\gimel }\Big )^{\omega _{i}}-1\Big )\frac{1}{\gimel }}\\ \\ {\frac{1+\gimel }{\gimel }\Big (1-\prod _{i=1}^{N}\Big (1-{\iota }_{i}(\frac{\gimel }{1+\gimel })\Big )^{\omega _{i}}\Big )}\\ \\ {\frac{1+\gimel }{\gimel }\Big (1-\prod _{i=1}^{N}\Big (1-{\phi }_{i}(\frac{\gimel }{1+\gimel })\Big )^{\omega _{i}}\Big )}\Bigg ), \end{array} \right\} \oplus \omega _{N+1}\daleth _{\sigma _{N+1}} \end{aligned}$$

$$\begin{aligned}= & \left\{ \begin{array}{c} \Bigg ({\Big ((1+\gimel )\prod _{i=1}^{N+1}\Big (\frac{\gimel {\tau }_{i}+1}{1+\gimel }\Big )^{\omega _{i}}-1\Big )\frac{1}{\gimel }}\\ \\ {\frac{1+\gimel }{\gimel }\Big (1-\prod _{i=1}^{N+1}\Big (1-{\iota }_{i}(\frac{\gimel }{1+\gimel })\Big )^{\omega _{i}}\Big )}\\ \\ {\frac{1+\gimel }{\gimel }\Big (1-\prod _{i=1}^{N+1}\Big (1-{\phi }_{i}(\frac{\gimel }{1+\gimel })\Big )^{\omega _{i}}\Big )}\Bigg ), \end{array} \right\} \end{aligned}$$

this implies that N+1 holds. Hence it is true for all n and it completes the proof. $\square$

#### Property 4.18

**Idempotency :**Let $\alpha _{\sigma _i}= \Big \{{\tau }_{\alpha _{\sigma _i}},{\iota }_{\alpha _{\sigma _i}},{\phi }_{\alpha _{\sigma _i}}\Big \}$ and $i \in N$ be a selection of *INS*s if all them $\alpha _{\sigma _i}$ are identical then

$$\begin{aligned} INWG_{SW}(\alpha _{\sigma _1},\alpha _{\sigma _2},\alpha _{\sigma _3}..., \alpha _{\sigma _n})=\alpha _{\sigma } \end{aligned}$$

#### Proof

let all the $\alpha _{\sigma _i}$ are identical and we know that

$$\begin{aligned} INWG_{SW}(\alpha _{\sigma _1},\alpha _{\sigma _2},\alpha _{\sigma _3}..., \alpha _{\sigma _n})= & \prod _{i=1}^{n}\alpha _{\sigma _{i}}^{\omega _{i}}\\= & \left\{ \begin{array}{c} \Bigg ({\Big ((1+\gimel )\prod _{i=1}^{n}\Big (\frac{\gimel {\tau }_{i}+1}{1+\gimel }\Big )^{\omega _{i}}-1\Big )\frac{1}{\gimel }}\\ \\ {\frac{1+\gimel }{\gimel }\Big (1-\prod _{i=1}^{n}\Big (1-{\iota }_{i}(\frac{\gimel }{1+\gimel })\Big )^{\omega _{i}}\Big )}\\ \\ {\frac{1+\gimel }{\gimel }\Big (1-\prod _{i=1}^{n}\Big (1-{\phi }_{i}(\frac{\gimel }{1+\gimel })\Big )^{\omega _{i}}\Big )}\Bigg ), \end{array} \right\} \\= & \left\{ \begin{array}{c} \Bigg ({\Big ((1+\gimel )\Big (\frac{\gimel {\tau }_{i}+1}{1+\gimel }\Big )^{\sum _{i=1}^{n}\omega _{i}}-1\Big )\frac{1}{\gimel }}\\ \\ {\frac{1+\gimel }{\gimel }\Big (1-\Big (1-{\iota }_{i}(\frac{\gimel }{1+\gimel })\Big )^{\sum _{i=1}^{n}\omega _{i}}\Big )}\\ \\ {\frac{1+\gimel }{\gimel }\Big (1-\Big (1-{\phi }_{i}(\frac{\gimel }{1+\gimel })\Big )^{\sum _{i=1}^{n}\omega _{i}}\Big )}\Bigg ), \end{array} \right\} \\= & \left\{ \begin{array}{c} \Bigg ({\Big ((1+\gimel )\Big (\frac{\gimel {\tau }_{i}+1}{1+\gimel }\Big )-1\Big )\frac{1}{\gimel }}\\ \\ {\frac{1+\gimel }{\gimel }\Big (1-\Big (1-{\iota }_{i}(\frac{\gimel }{1+\gimel })\Big )\Big )}\\ \\ {\frac{1+\gimel }{\gimel }\Big (1-\Big (1-{\phi }_{i}(\frac{\gimel }{1+\gimel })\Big )\Big )}\Bigg ), \end{array} \right\} \\= & \alpha _{\sigma }. \end{aligned}$$

$$\square$$

#### Property 4.19

**Monotonicity:** Let $\alpha _{\sigma _i}= \Big \{{\tau }_{\alpha _{\sigma _i}},{\iota }_{\alpha _{\sigma _i}},{\phi }_{\alpha _{\sigma _i}}\Big \}$ and $\alpha _{\sigma _i'}= \Big \{{\tau }_{\alpha _{\sigma _i'}},{\iota }_{\alpha _{\sigma _i'}},{\phi }_{\alpha _{\sigma _i'}}\Big \}$ $i, i'={1,2,3...,n}$ be a assemblages of *INS* so as $\alpha _{\sigma _i}\subseteq \alpha _{\sigma _i'}$ then,

$$\begin{aligned} INWG_{SW}(\alpha {\sigma _1},\alpha _{\sigma _2},\alpha _{\sigma _3} ..., \alpha _{\sigma _n}) \le INWG_{SW}(\alpha _{\sigma _1'},\alpha _{\sigma _2'},\alpha _{\sigma _3'} ..., \alpha _{\sigma _n'}) \end{aligned}$$

#### Proof

As $\alpha _{\sigma _i}\subseteq \alpha _{\sigma _i'}$ Thus ${\tau }_{\alpha _{\sigma _i}}\le {\tau }_{\alpha _{\sigma _i'}}$ , ${\iota }_{\alpha _{\sigma _i}}\le {\iota }_{\alpha _{\sigma _i'}}$ and ${\phi }_{\alpha _{\sigma _i}}\ge {\phi }_{\alpha _{\sigma _i'}}$ this implies that

$$\begin{aligned} {\Big ((1+\gimel )\prod _{i=1}^{n}\Big (\frac{\gimel {\tau }_{i}+1}{1+\gimel }\Big )^{\omega _{i}}-1\Big )\frac{1}{\gimel }}\ge {\Big ((1+\gimel )\prod _{i'=1}^{n}\Big (\frac{\gimel {\tau }_{i'}+1}{1+\gimel }\Big )^{\omega _{i'}}-1\Big )\frac{1}{\gimel }}, \end{aligned}$$

$$\begin{aligned} {\Big ((1+\gimel )\prod _{i=1}^{n}\Big (\frac{\gimel {\tau }_{i}+1}{1+\gimel }\Big )^{\omega _{i}}-1\Big )\frac{1}{\gimel }}\le {\Big ((1+\gimel )\prod _{i'=1}^{n}\Big (\frac{\gimel {\imath }_{i'}+1}{1+\gimel }\Big )^{\omega _{i'}}-1\Big )\frac{1}{\gimel }}, \end{aligned}$$

$$\begin{aligned} {\frac{1+\gimel }{\gimel }\Big (1-\prod _{i=1}^{n}\Big (1-{\phi }_{i}(\frac{\gimel }{1+\gimel })\Big )^{\omega _{i}}\Big )}\le {\frac{1+\gimel }{\gimel }\Big (1-\prod _{i'=1}^{n}\Big (1-{\phi }_{i'}(\frac{\gimel }{1+\gimel })\Big )^{\omega _{i'}}\Big )}, \end{aligned}$$

this implies that

$$\begin{aligned} \left\{ \begin{array}{c} \Bigg ({\Big ((1+\gimel )\prod _{i=1}^{n}\Big (\frac{\gimel {\tau }_{i}+1}{1+\gimel }\Big )^{\omega _{i}}-1\Big )\frac{1}{\gimel }}\\ \\ {\frac{1+\gimel }{\gimel }\Big (1-\prod _{i=1}^{n}\Big (1-{\iota }_{i}(\frac{\gimel }{1+\gimel })\Big )^{\omega _{i}}\Big )}\\ \\ {\frac{1+\gimel }{\gimel }\Big (1-\prod _{i=1}^{n}\Big (1-{\phi }_{i}(\frac{\gimel }{1+\gimel })\Big )^{\omega _{i}}\Big )}\Bigg ), \end{array} \right\} \le \left\{ \begin{array}{c} \Bigg ({\Big ((1+\gimel )\prod _{i'=1}^{n}\Big (\frac{\gimel {\tau }_{i'}+1}{1+\gimel }\Big )^{\omega _{i'}}-1\Big )\frac{1}{\gimel }}\\ \\ {\frac{1+\gimel }{\gimel }\Big (1-\prod _{i'=1}^{n}\Big (1-{\iota }_{i'}(\frac{\gimel }{1+\gimel })\Big )^{\omega _{i'}}\Big )}\\ \\ {\frac{1+\gimel }{\gimel }\Big (1-\prod _{i'=1}^{n}\Big (1-{\phi }_{i'}(\frac{\gimel }{1+\gimel })\Big )^{\omega _{i'}}\Big )}\Bigg ), \end{array} \right\} \end{aligned}$$

Hence

$$\begin{aligned} INWG_{SW}(\alpha _{\sigma _1},\alpha _{\sigma _2},\alpha _{\sigma _3} ..., \alpha _{\sigma _n}) \le INWG_{SW}(\alpha _{\sigma _1'},\alpha _{\sigma _2'},\alpha _{\sigma _3'} ..., \alpha _{\sigma _n'}) \end{aligned}$$

$$\square$$

#### Property 4.20

**Boundedness:** Let $\alpha _{\sigma _i}= \Big \{{\tau }_{\alpha _{\sigma _i}},{\iota }_{\alpha _{\sigma _i}},{\phi }_{\alpha _{\sigma _i}}\Big \}$ and $i \in N$ be a assemblages of *INS* so as $\alpha _{\sigma A}=$ max $\alpha _{\sigma _i}$ and $\alpha _{\sigma _B}=$ min $\alpha _{\sigma _i}$ then,

$$\begin{aligned} \alpha _{\sigma _B}\le INWG_{SW}(\alpha _{\sigma _1},\alpha _{\sigma _2},\alpha _{\sigma _3} ..., \alpha _{\sigma _n})\le \alpha _{\sigma A} \end{aligned}$$

#### Proof

The proof is straight forward. $\square$

#### Definition 4.21

Let $\alpha _{\sigma _i}= \Big \{{\tau }_{\alpha _{\sigma _i}},{\iota }_{\alpha _{\sigma _i}},{\phi }_{\alpha _{\sigma _i}}\Big \}$ and $i \in N$ be a selection of *INS*s and the $INHWA_{SW}$ $INOWG_{SW}: INS^{N}\rightarrow INS$ is defined as

$$\begin{aligned} INOWG_{SW}(\alpha _{\sigma _1},\alpha _{\sigma _2},\alpha _{\sigma _3} ..., \alpha _{\sigma _n})=\prod _{i=1}^{n}\alpha ^{\omega _{i}}_{\sigma _{\delta (i)}} \end{aligned}$$

with n dimensions so as ith HWV is $\alpha _{\sigma _i}$ as a result, by the overall order $\alpha _{\sigma _1}\ge \alpha _{\sigma _2} \ge \alpha _{\sigma _3} \ge ...\ge \alpha _{\sigma _n}$ and $0\le \omega _{i}\le 1, \sum _{1}^{n}\omega _{i}=1$ and $\omega _{i}$ signify W-V.

#### Theorem 4.22

Let $\alpha _{\sigma _{i}}=\Big \{{\tau }_{\alpha _{\sigma _i}},{\iota }_{\alpha _{\sigma _i}},{\phi }_{\alpha _{\sigma _i}}\Big \}$ be a selection of *INS*s, where $i\in N$ then $INWG_{SW}$ is defined as:

$$\begin{aligned} INOWG_{SW}(\alpha _{\sigma _1},\alpha _{\sigma _2},\alpha _{\sigma _3}..., \alpha _{\sigma _n})= & \prod _{i=1}^{n}\alpha ^{\omega _{i}}_{\sigma _{\Delta (i)}}\\= & \left\{ \begin{array}{c} \Bigg ({\Big ((1+\gimel )\prod _{i=1}^{n}\Big (\frac{\gimel {\tau }_{\Delta (i)}+1}{1+\gimel }\Big )^{\omega _{i}}-1\Big ) \frac{1}{\gimel }}\\ \\ {\frac{1+\gimel }{\gimel }\Big (1-\prod _{i=1}^{n}\Big (1-{\iota }_{\Delta (i)}(\frac{\gimel }{1+\gimel })\Big )^{\omega _{i}}\Big )}\\ \\ {\frac{1+\gimel }{\gimel }\Big (1-\prod _{i=1}^{n}\Big (1-{\phi }_{\Delta (i)}(\frac{\gimel }{1+\gimel })\Big )^{\omega _{i}}\Big )}\Bigg ), \end{array} \right\} \end{aligned}$$

with n dimensions so as $\alpha _{\sigma _i}$ is the ith HWV consequently the total order $\alpha _{\sigma _1}\ge \alpha _{\sigma _2} \ge \alpha _{\sigma _3} \ge ...\ge \alpha _{\sigma _n}$ and $0\le \omega _{i}\le 1, \sum _{1}^{n}\omega _{i}=1$ and $\omega _{i}$ signify W-V.

#### Proof

The proof relates to the $INWA_{SW}$ operator above. Hence, we avoid it. $\square$

#### Property 4.23

**Idempotency :**Let $\alpha _{\sigma _i}= \Big \{{\tau }_{\alpha _{\sigma _i}},{\iota }_{\alpha _{\sigma _i}},{\phi }_{\alpha _{\sigma _i}}\Big \}$ and $i \in N$ be a selection of *INS*s if all them $\alpha _{\sigma _i}$ are identical then

$$\begin{aligned} INOWG_{SW}(\alpha _{\sigma _1},\alpha _{\sigma _2},\alpha _{\sigma _3} ..., \alpha _{\sigma _n})=\alpha _{\sigma } \end{aligned}$$

#### Property 4.24

**Monotonicity:** Let $\alpha _{\sigma _i}= \Big \{{\tau }_{\alpha _{\sigma _i}},{\iota }_{\alpha _{\sigma _i}},{\phi }_{\alpha _{\sigma _i}}\Big \}$ and $\alpha _{\sigma _{i'}}= \Big \{{\tau }_{\alpha _{\sigma _i'}},{\iota }_{\alpha _{\sigma _i'}},{\phi }_{\alpha _{\sigma _i'}}\Big \}$ $i, i'={1,2,3...,n}$ be a assemblages of *INS* so as $\alpha _{\sigma _i}\subseteq \alpha _{\sigma _i'}$ then,

$$\begin{aligned} INOWG_{SW}(\alpha _{\sigma _1},\alpha _{\sigma _2},\alpha _{\sigma _3} ..., \alpha _{\sigma _n}) \le INOWG_{SW}(\alpha _{\sigma _1'},\alpha _{\sigma _2'},\alpha _{\sigma _3'} ..., \alpha _{\sigma _n'}) \end{aligned}$$

#### Property 4.25

**Boundedness:** Let $\alpha _{\sigma _i}= \Big \{{\tau }_{\alpha _{\sigma _i}},{\iota }_{\alpha _{\sigma _i}},{\phi }_{\alpha _{\sigma _i}}\Big \}$ and $i \in N$ be a assemblages of *INS* so as $\alpha _{\sigma A}=$ max $\alpha _{\sigma _i}$ and $\alpha _{\sigma _B}=$ min $\alpha _{\sigma _i}$ then,

$$\begin{aligned} \alpha _{\sigma _B}\le INOWG_{SW}(\alpha _{\sigma _1},\alpha _{\sigma _2},\alpha _{\sigma _3} ..., \alpha _{\sigma _n})\le \alpha _{\sigma A} \end{aligned}$$

#### Definition 4.26

Let $\alpha _{\sigma _i}= \Big \{{\tau }_{\alpha _{\sigma _i}},{\iota }_{\alpha _{\sigma _i}},{\phi }_{\alpha _{\sigma _i}}\Big \}$ and $i \in N$ be a selection of *INS*s and the $INHWA_{SW}$ $INHWG_{SW}:INS^{N}\rightarrow INS$ is defined as

$$\begin{aligned} INHWG_{SW}(\alpha _{\sigma _1},\alpha _{\sigma _2},\alpha _{\sigma _3} ..., \alpha _{\sigma _n})=\prod _{i=1}^{n}(\alpha '_{\sigma _{\delta (i)}})^{\lambda _{i}} \end{aligned}$$

with n dimensions so as ith HWV is $\alpha _{\sigma _{\Delta (i)}}$ and $\alpha '_{\sigma _i}=\alpha ^{n{\lambda _{i}}}_{\sigma _i},(i\in N)$, $0\le \omega _{i}\le 1, \sum _{1}^{n}\omega _{i}=1$ and $\omega _{i}$ signify W-V also ${\lambda _{i}}$ is the associated W-V $0\le \lambda _{i}\le 1, \sum _{1}^{n}\lambda _{i}=1$ and the B-C is n

#### Theorem 4.27

Let $\alpha _{\sigma _{i}}=\Big \{{\tau }_{\alpha _{\sigma _i}},{\iota }_{\alpha _{\sigma _i}},{\phi }_{\alpha _{\sigma _i}}\Big \}$ be a selection of *INS*s, where $i\in N$ then $INHWG_{SW}$ is defined as:

$$\begin{aligned} INHWG_{SW}(\alpha _{\sigma _1},\alpha _{\sigma _2},\alpha _{\sigma _3}..., \alpha _{\sigma _n})= & \prod _{i=1}^{n}(\alpha '_{\sigma _{\delta (i)}})^{\lambda _{i}}\\= & \left\{ \begin{array}{c} \Bigg ({\Big ((1+\gimel )\prod _{i'=1}^{n}\Big (\frac{\gimel {\tau }_{i'}+1}{1+\gimel }\Big )^{\lambda _{i'}}-1\Big )\frac{1}{\gimel }},\\ \\ {\frac{1+\gimel }{\gimel }\Big (1-\prod _{i'=1}^{n}\Big (1-{\iota }_{i'}(\frac{\gimel }{1+\gimel })\Big )^{\lambda _{i'}}\Big )},\\ \\ {\frac{1+\gimel }{\gimel }\Big (1-\prod _{i'=1}^{n}\Big (1-{\phi }_{i'}(\frac{\gimel }{1+\gimel })\Big )^{\lambda _{i'}}\Big )}\Bigg ), \end{array} \right\} \end{aligned}$$

with n dimensions so as ith HWV is $\alpha _{\sigma _{\Delta (i)}}$ and $\alpha '_{\sigma _i}=\alpha ^{n{\lambda _{i}}}_{\sigma _i},(i\in N)$ , $0\le \omega _{i}\le 1, \sum _{1}^{n}\omega _{i}=1$ and $\omega _{i}$ signify W-V, also ${\lambda _{i}}$ is the associated W-V, $0\le \lambda _{i}\le 1, \sum _{1}^{n}\lambda _{i}=1$ and the B-C is n

#### Proof

The proof relates to the $INWG_{SW}$ operator above. Hence, we avoid it. $\square$

#### Property 4.28

**Idempotency:** Let $\alpha _{\sigma _i}= \Big \{{\tau }_{\alpha _{\sigma _i}},{\iota }_{\alpha _{\sigma _i}},{\phi }_{\alpha _{\sigma _i}}\Big \}$, $i \in N$ be a selection of *INS*s if all them $\alpha _{\sigma _i}$ are identical, then

$$\begin{aligned} INHWG_{SW}(\alpha _{\sigma _1},\alpha _{\sigma _2},\alpha _{\sigma _3} ..., \alpha _{\sigma _n})=\alpha _{\sigma } \end{aligned}$$

#### Property 4.29

**Monotonicity:** Let $\alpha _{\sigma _i}= \Big \{{\tau }_{\alpha _{\sigma _i}},{\iota }_{\alpha _{\sigma _i}},{\phi }_{\alpha _{\sigma _i}}\Big \}$ and $\alpha _{\sigma _{i'}}= \Big \{{\tau }_{\alpha _{\sigma _i'}},{\iota }_{\alpha _{\sigma _i'}},{\phi }_{\alpha _{\sigma _i'}}\Big \}$ $i, i'={1,2,3...,n}$ be a assemblages of *INS* so as $\alpha _{\sigma _i}\subseteq \alpha _{\sigma _i'}$ then,

$$\begin{aligned} INHWG_{SW}(\alpha _{\sigma _1},\alpha _{\sigma _2},\alpha _{\sigma _3} ..., \alpha _{\sigma _n}) \le INHWG_{SW}(\alpha _{\sigma _1'},\alpha _{\sigma _2'},\alpha _{\sigma _3'} ..., \alpha _{\sigma _n'}) \end{aligned}$$

#### Property 4.30

**Boundedness:** Let $\alpha _{\sigma _i}= \Big \{{\tau }_{\alpha _{\sigma _i}},{\iota }_{\alpha _{\sigma _i}},{\phi }_{\alpha _{\sigma _i}}\Big \}$ and $i \in N$ be a assemblages of *INS* so as $\alpha _{\sigma A}=$ max $\alpha _{\sigma _i}$ and $\alpha _{\sigma _B}=$ min $\alpha _{\sigma _i}$ then,

$$\begin{aligned} \alpha _{\sigma _B}\le INHWG_{SW}(\alpha _{\sigma _1},\alpha _{\sigma _2},\alpha _{\sigma _3} ..., \alpha _{\sigma _n})\le \alpha _{\sigma A} \end{aligned}$$

## $IN_{SW}$ operator for federated edge-AI enabled autonomous drone swarms for real-time leak detection in natural gas pipelines

These INS values are organized into a swarm wide decision matrix and it is summed with the proposed INS-SW operators. A ranked list of the suspected leak locations is then transmitted back to a central coordinator or autonomous inspection missions can be triggered thus closing distributed sensing, federated decision-making and physical response loop. The average and geometric AgOs, which we previously proposed as a solution to uncertainty issues in autonomous system DM, are described in the next section. We provide an example of a real-time pipeline leak detection scenario where these operators might be applied to support sensor data fusion and DM in order to demonstrate their practicality. In this examination, we may take as a set of detection alternatives, $\gimel = \left\langle \gimel _1, \gimel _2, \gimel _3, \dots , \gimel _n \right\rangle$, where and $\gimel$ and $\gimel _i$, denote potential leak locations or anomaly states detected by the drone swarm. Moreover, there is a set of sensor and environmental indicators $\digamma$ that we assume to be a numerical set of environmental indicators $\digamma = \{\digamma _1, \digamma _2, \digamma _3, \dots , \digamma _n\}$. Every indicator has a weight-vector $\omega = \{\omega _1, \omega _2, \dots , \omega _n\}$,to convey its relative importance in the process of leak localization and anomaly detection. These weights must meet the standard requirements, which state that each weight must fall between 0 and 1 and that the total of all the weights must equal 1 in order to provide a suitable and balanced weighting scheme. The framework improves the accuracy and dependability of autonomous pipeline monitoring by enabling the combination of heterogeneous sensor data and accounting for the varying significance of each detection criterion.

**Figure:**
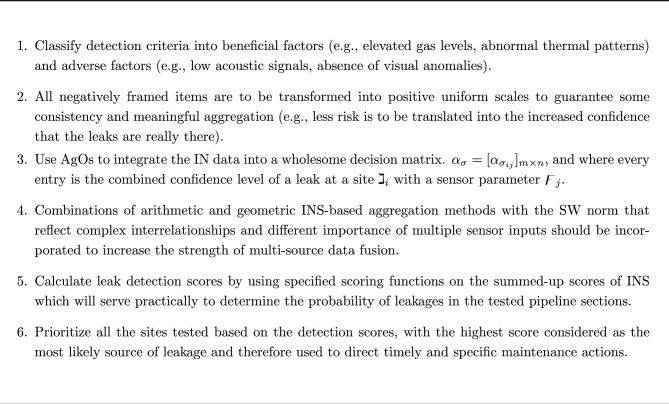
Algorithm

**Fig. 1:**
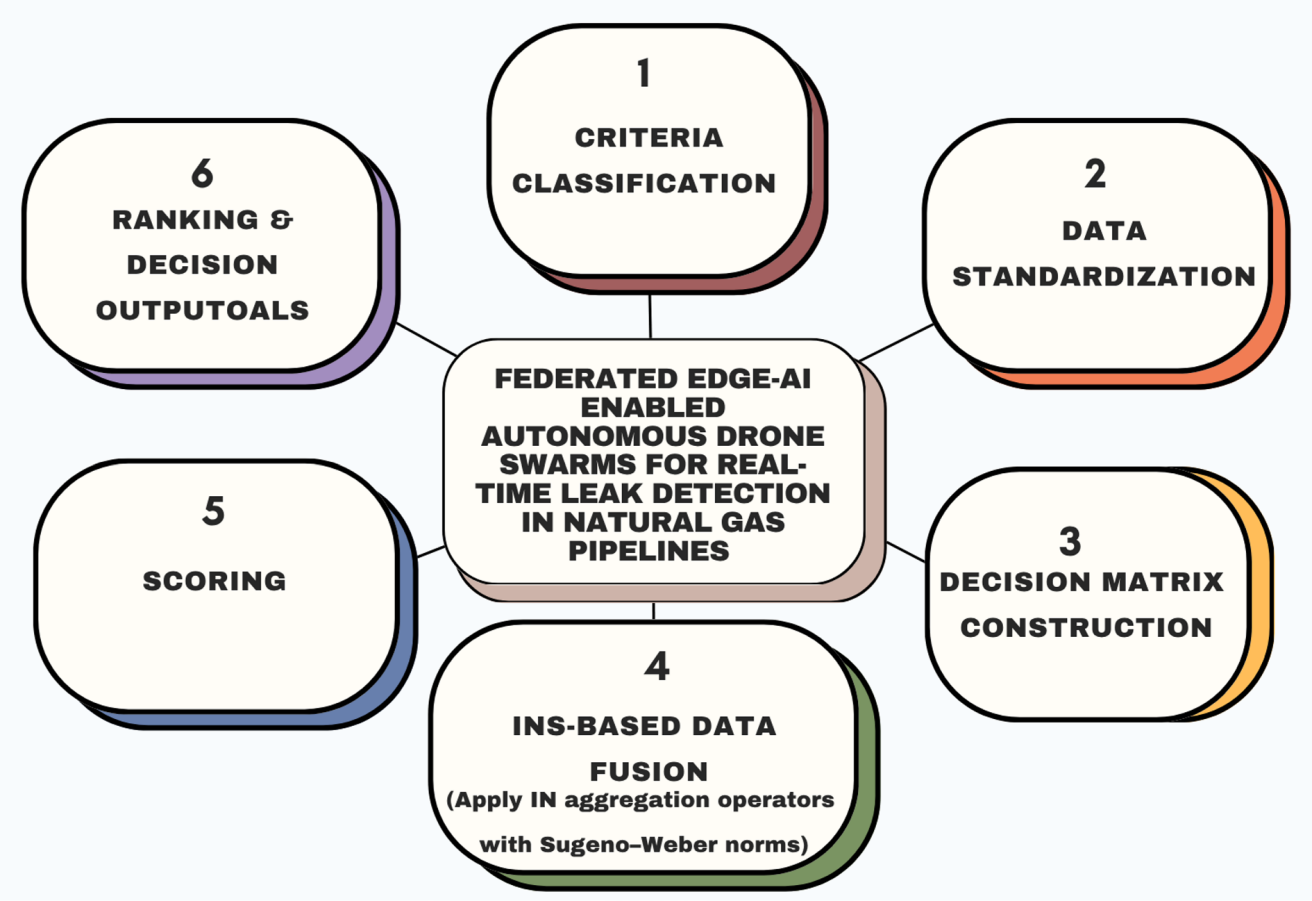
Flow chart of algorithm.

The flowchart of the algorithm is given in Fig. 1.

## Numerical problem

This section outlines a case study that aims at an assessment of sophisticated pipeline monitoring strategies with a specific concentration on fuzzy DM in unpredictable and risky infrastructure settings. The four alternatives of technology have also been contrasted, and they are quantum-enhanced federated learning of drone swarms, AI-powered repair drones (Self-Healing Pipelines), Digital Twin-Enabled Pipelines with Real-Time Drone Feedback, and Hybrid Underwater-Aerial Drone Swarms of Sub-sea and Land Pipelines. In order to compare them, four key qualities are considered, the core of the present pipeline integrity systems: Federated Edge AI with Privacy-Preserving Learning, Autonomous Swarm Self-Organization and Self-Healing Network, Multi-Sensor Fusion AI to Ultra-precise Leak Detection and Blockchain-Secured Federated Updates with Cyber Resilience. These qualities are technological, operational and security aspects that characterize the possibility and desirability of the pipeline observation systems. The suggested framework is a good way to represent the complicated stratification of implementing intelligent technologies in uncertain conditions of work, including sensor noise, communication latency, hostile environment, or possible cyber interference. Accuracy of detection, security of the network, and the overall reliability of the system are some of the most urgent that have a heavy influence on the long-term adoption and sustainability. To illustrate the functionality of the proposed framework, an elaborate numerical case study that is founded on a pipeline leak detection scenario is given. Several pipeline sections are analyzed through decision attributes based on the results of drone observations, such as the intensity of gas, pressure variation, thermal anomaly, and sensor reliability. The IN decision-matrix is built with the help of the outputs of the edge-AI, and then the aggregation of the decision-matrix is accomplished on the basis of the offered Sugeno-Weber operators. The end results are final classifications of pipeline segments, which is a clear demonstration of the capability of the framework to discriminate the areas that are prone to leakage in cases of uncertainty. The sensitivity analysis in the light of Sugeno-Weber control parameter also attests the solidity and stability of the decision outcomes. Quantum-Enhanced Federated Learning can perform better in the example of noisy data but may be more computationally or hardware demanding, as an instance. Self-healing repair drones offer instant solutions to faults repair, although with some problems concerning the energy efficiency and lifetime of wear and tear. Digital Twin systems offer predictive analytic solutions and high fidelity models yet the system requires a significant amount of accuracy in calibration and streamlined data feeds. Hybrid underwater-Aerial swarms are capable of covering the submerged infrastructures as well as the surface ones but would need to confront very crucial coordination, communication and logistical constraints. Besides the technical constraints, the perceptions of the stakeholders are a factor that determines the implementation of technology. Even the and excellent performing systems can be opposed due to privacy or cost of preliminary investment or difficulty of installation in distant locations. Using a DM framework based on fuzzy logic, it is possible to transform qualitative judgments (concerning privacy, energy, security, and autonomy) made qualitatively by experts into quantitative factors in a systematic fashion. This is a systematic analysis that displays trade-offs and assists in establishing strategic priorities of investment and deployment to decision-makers. Through the application of fuzzy DM techniques i.e. those that combine both INS and linguistic variables and membership functions, this paper will convert the subjective ratings into quantifiable ones. The resulting model offers practical information on what monitoring strategy will provide the most desirable balance between performance, security, resilience and operational practicality in the uncertainty scenario. The results are, therefore, a strategic guide to policy makers, engineers and operators on how they can make investments, deploy and allocate resources to achieve a sustainable, resilient and high-assurance system of monitoring the pipeline.

The diverse technological strategies that can be adopted by energy regulators, engineers, or pipeline operators to enhance the monitoring and maintenance of natural gas infrastructure include quantum-enhanced federated learning for drone swarms, repair drones capable of self-healing pipelines, digital twin-based pipelines with real-time drone feedback, and hybrid underwater-aerial drone swarms for both sub-sea and terrestrial networks. These options are considered in the evaluation of federated edge-enabled autonomous drone swarms for real-time leak detection in natural gas pipelines, where critical attributes such as privacy-preserving federated learning, autonomous swarm coordination with self-healing capabilities, multi-sensor fusion for highly accurate leak localization, and blockchain-secured federated updates with strong cyber resilience are essential for achieving dependable, secure, and precise operation:

**Quantum-enhanced Federated Learning of Drone Swarms** $\gimel _{1}$: A Drone-based Programming Language. This alternative uses quantum-assisted optimization as part of distributed learning to enable drone swarms to process large-scale information with superior convergence and capability in making decisions. It facilitates complicated leak detection requirements, however, it is resource-demanding and needs unique infrastructure.

Self-healing pipelines are powered by AI guided repair drones forming a repair repair loop whereby new repairs occur infrequently, and the pipe can automatically mend the damage itself. **AI Powered Repair Drones (Self-Healing Pipelines)** $\gimel _{2}$: This concept adopts maintenance drones that are to deal with the above-ground repair by themselves will notice and fix spillages, forming self-healing pipeline communities. It reduces downtime and operational risks and prolongs the life of infrastructure although it comes with high technical complexity and regulatory issues.

**Digital Twin Enabled Pipelines with Real-Time Drone Feedback** $\gimel _{3}$: A digital twin is an equivalent virtual simulation of a pipeline that is constantly updated with the information taken by drones. It allows predictive maintenance, proactive risk analysis and system wide monitoring. Nonetheless, it needs sophisticated data integration platforms and high levels of cybersecurity.

**Hybrid Underwater-Aerial Drone Swarms for Sub sea and Land Pipelines** $\gimel _{4}$: This approach synchronizes drones in the air and underwater for total coverage of terrestrial and subsea pipelines This method organizes drone initiatives on the air and sea to cover terrestrial and underwater pipes. It has a strong monitoring capability under a variety of conditions, but has difficulty in cross-domain communications, synchronization, and durability.

These alternatives are evaluated on the basis of a list of key features:

**Federated Edge AI with Privacy Preserving Learning** $\digamma _{1}$: Determines the capacity of each approach to secure and distributed learning and guard sensitive operating data. Digital twins INS and federated frameworks are working effectively whereas the hybrid swarms might experience gaps in integration.

**Autonomous Swarm Self-Organization and Self-Healing Networks** $\digamma _{2}$: Tests the ability of drone swarms to restructure and ensure continuity in operation even in unfavorable scenarios. The resilience scores of repair drones and hybrid swarms are usually higher.

**Multi-Sensor Fusion AI for Ultra-Precise Leak Detection** $\digamma _{3}$: Determines the accuracy of the leak localization by the means of the high-level sensor integration. Quantum-enhanced federated learning and digital twin INS are more accurate, whereas repair drones pay more attention to correctional measures.

**Blockchain Secured Federated Updates and Cyber Resilience** $\digamma _{4}$: Should be able to provide security and integrity of joint modification to the whole decision structure. The systems based on blockchain provide a high level of protection and can be used in federated swarm and digital twins.

Accordingly, the set of alternatives is defined as $\gimel =\{\gimel _{1},\gimel _{2},\gimel _{3},\gimel _{4}\}$, and the set of attributes is defined as $\digamma =\{\digamma _{1},\digamma _{2},\digamma _{3},\digamma _{4}\}$. The weight vector is given by $\omega =\{\omega _{1}=0.38,\ \omega _{2}=0.47,\ \omega _{3}=0.15\}$, reflecting the relative importance assigned by expert groups (e.g., regulators, engineers, and operators). Additionally, we define $\lambda =[0.15,0.25,0.25,0.35]$ and set $\alpha =2$. These correlated weight vectors are the interdependence of attributes in the DM process.

**Case Study:** Federated Edge-AI Enabled Autonomous Drone Swarms for Real-Time Leak Detection in Natural Gas Pipelines. In the case study, the factors influencing the technology adoption are observed by comparing the four strategic options, such as Quantum Enhanced Federated Learning to Drone Swarms, AI-Powered Repair Drones (Self-Healing Pipelines), Digital Twin Enabled Pipelines with Real-Time Drone Feedback, and Hybrid Underwater-Aerial Drone Swarms to Subsea and Land Pipelines, to the factors. A fuzzy DM model with INS and the operation of SW is implemented to control the imprecision and ambiguity of the expert rating, risks of operations, and trade-offs involving technology. Finally, but not least, the research at hand assists the stakeholders in developing effective approaches to enhance the security, resilience, and performance of the pipeline monitoring systems by pointing out how various options could be incorporated in the changing environment of the energy infrastructure. The proposed model will differentiate Federated Edge AI with Privacy Preserving Learning, Autonomous Swarm Self-Organization and Self-Healing Networks, Multi-Sensor Fusion AI in Leak Detection of Ultra-Precision, and Blockchain-secured Federated Updates and Cyber Resilience in comparison to the traditional evaluation methodology and play a role in the evidence-based policy-making in critical infrastructure protection.

### Numerical illustrations

This section presents the assessment matrix of a numerical analysis that has been adapted to apply fuzzy DM methods to the selection of advanced pipeline monitoring and maintenance strategies. More specifically, the framework considers four technological options Quantum Enhanced Federated Learning for Drone Swarms, AI Powered Repair Drones (Self-Healing Pipelines), Digital Twin Enabled Pipelines with Real-Time Drone Feedback, and Hybrid Underwater-Aerial Drone Swarms for Subsea and Land Pipelines and evaluates them against INS is the key criterion of Federated Edge AI with Privacy-Preserving Learning, Autonomous Swarm Self-Organization and Self-Healing Networks, Multi-Sensor Fusion AI for Ultra-Precise Leak Detection, and Blockchain-Secured Federated Updates and Cyber Resilience using INS.

Step 1: Decision matrices by the expert $\yen _{1}$ ,expert $\yen _{2}$ and expert $\yen _{3}$ In Table 1, 2, and 3 respectively.

**Table 1:** D-Mx by the expert $\yen _{1}$.

$\alpha _{\sigma _{ij}}$	$\digamma _{1}$	$\digamma _{2}$	$\digamma _{3}$	$\digamma _{4}$
$\gimel _{1}$	$(0.3,\ 0.2,\ 0.6)$	$(0.4,\ 0.3,\ 0.2)$	$(0.4,\ 0.8,\ 0.4)$	$(0.2,\ 0.3,\ 0.2)$
$\gimel _{2}$	$(0.4,\ 0.2,\ 0.1)$	$(0.2,\ 0.3,\ 0.4)$	$(0.2,\ 0.2,\ 0.5)$	$(0.2,\ 0.4,\ 0.7)$
$\gimel _{3}$	$(0.5,\ 0.2,\ 0.3)$	$(0.4,\ 0.5,\ 0.5)$	$(0.3,\ 0.8,\ 0.6)$	$(0.4,\ 0.6,\ 0.4)$
$\gimel _{4}$	$(0.8,\ 0.6,\ 0.3)$	$(0.4,\ 0.7,\ 0.4)$	$(0.6,\ 0.7,\ 0.3)$	$(0.5,\ 0.5,\ 0.4)$

**Table 2:** D-Mx by the expert $\yen _{2}$.

$\alpha _{\sigma _{ij}}$	$\digamma _{1}$	$\digamma _{2}$	$\digamma _{3}$	$\digamma _{4}$
$\gimel _{1}$	$(0.4,\ 0.5,\ 0.2)$	$(0.5,\ 0.6,\ 0.2)$	$(0.3,\ 0.3,\ 0.3)$	$(0.2,\ 0.5,\ 0.4)$
$\gimel _{2}$	$(0.2,\ 0.3,\ 0.3)$	$(0.3,\ 0.9,\ 0.5)$	$(0.4,\ 0.3,\ 0.5)$	$(0.3,\ 0.4,\ 0.2)$
$\gimel _{3}$	$(0.2,\ 0.2,\ 0.2)$	$(0.4,\ 0.4,\ 0.4)$	$(0.1,\ 0.9,\ 0.2)$	$(0.4,\ 0.3,\ 0.5)$
$\gimel _{4}$	$(0.3,\ 0.5,\ 0.2)$	$(0.2,\ 0.5,\ 0.4)$	$(0.2,\ 0.5,\ 0.4)$	$(0.4,\ 0.9,\ 0.4)$

**Table 3:** D-Mx by the expert $\yen _{3}$.

$\alpha _{\sigma _{ij}}$	$\digamma _{1}$	$\digamma _{2}$	$\digamma _{3}$	$\digamma _{4}$
$\gimel _{1}$	$(0.4,\ 0.9,\ 0.4)$	$(0.2,\ 0.3,\ 0.3)$	$(0.5,\ 0.8,\ 0.4)$	$(0.4,\ 0.5,\ 0.4)$
$\gimel _{2}$	$(0.3,\ 0.4,\ 0.4)$	$(0.4,\ 0.7,\ 0.5)$	$(0.2,\ 0.2,\ 0.3)$	$(0.2,\ 0.7,\ 0.2)$
$\gimel _{3}$	$(0.3,\ 0.9,\ 0.5)$	$(0.3,\ 0.6,\ 0.2)$	$(0.2,\ 0.4,\ 0.5)$	$(0.4,\ 0.4,\ 0.4)$
$\gimel _{4}$	$(0.2,\ 0.2,\ 0.3)$	$(0.5,\ 0.5,\ 0.3)$	$(0.4,\ 0.8,\ 0.3)$	$(0.5,\ 0.3,\ 0.3)$

Step 2: Normalization is not done because these criteria are of the benefit type.
Step 3: In Table 4 by using $INWA_{SW}$ AgO aggregate all individuals’ INS decision matrices into a collective INS D-Matrix.

**Table 4:** Aggregate Decision matrices by use the $INWA_{SW}$ operator.

$\alpha _{\sigma _{ij}}$	$\digamma _{1}$	$\digamma _{2}$	$\digamma _{3}$	$\digamma _{4}$
$\gimel _{1}$	$(0.361,\ 0.443,\ 0.393)$	$(0.391,\ 0.394,\ 0.224)$	$(0.393,\ 0.597,\ 0.364)$	$(0.253,\ 0.415,\ 0.314)$
$\gimel _{2}$	$(0.308,\ 0.281,\ 0.234)$	$(0.288,\ 0.577,\ 0.459)$	$(0.274,\ 0.233,\ 0.446)$	$(0.236,\ 0.467,\ 0.368)$
$\gimel _{3}$	$(0.353,\ 0.332,\ 0.307)$	$(0.376,\ 0.487,\ 0.382)$	$(0.208,\ 0.717,\ 0.417)$	$(0.400,\ 0.436,\ 0.434)$
$\gimel _{4}$	$(0.513,\ 0.450,\ 0.263)$	$(0.361,\ 0.576,\ 0.374)$	$(0.424,\ 0.649,\ 0.334)$	$(0.466,\ 0.564,\ 0.374)$

Step 4(a): Utilizing the $INWA_{SW}$ and $INWG_{SW}$ operators in Table 5, integrate the qualities for every alternative.

**Table 5:** Using $INWA_{SW}$ and $INWG_{SW}$ operator.

$\alpha _{\sigma _{i}}$	$INWA_{SW}$	$INWG_{SW}$
$\gimel _{1}$	$(0.362,\ 0.441,\ 0.284)$	$(0.359,\ 0.447,\ 0.289)$
$\gimel _{2}$	$(0.277,\ 0.445,\ 0.413)$	$(0.276,\ 0.466,\ 0.418)$
$\gimel _{3}$	$(0.347,\ 0.501,\ 0.391)$	$(0.342,\ 0.514,\ 0.392)$
$\gimel _{4}$	$(0.411,\ 0.574,\ 0.354)$	$(0.408,\ 0.577,\ 0.355)$

Step 4(b(i)): Rearranged matrix of decisions Using Definition 2.5 in Table 6.

**Table 6:** Rearranged matrix.

$\alpha _{\sigma _{ij}}$	$\digamma _{1}$	$\digamma _{2}$	$\digamma _{3}$	$\digamma _{4}$
$\gimel _{1}$	$(0.391,\ 0.394,\ 0.224)$	$(0.253,\ 0.415,\ 0.314)$	$(0.361,\ 0.443,\ 0.393)$	$(0.393,\ 0.597,\ 0.364)$
$\gimel _{2}$	$(0.308,\ 0.281,\ 0.234)$	$(0.274,\ 0.233,\ 0.446)$	$(0.236,\ 0.467,\ 0.368)$	$(0.288,\ 0.577,\ 0.459)$
$\gimel _{3}$	$(0.353,\ 0.332,\ 0.307)$	$(0.400,\ 0.436,\ 0.434)$	$(0.376,\ 0.487,\ 0.382)$	$(0.208,\ 0.717,\ 0.417)$
$\gimel _{4}$	$(0.513,\ 0.450,\ 0.263)$	$(0.466,\ 0.564,\ 0.374)$	$(0.424,\ 0.649,\ 0.334)$	$(0.361,\ 0.576,\ 0.374)$

Step 4(b(ii)): Integrate the attributes for each alternative using $INOWA_{SW}$ and $INOWG_{SW}$ operator in Table 7.

**Table 7:** using $INOWA_{SW}$ and $INOWG_{SW}$ operator.

$\alpha _{\sigma _{i}}$	$INOWA_{SW}$	$INOWG_{SW}$
$\gimel _{1}$	$(0.318,\ 0.452,\ 0.329)$	$(0.314,\ 0.457,\ 0.332)$
$\gimel _{2}$	$(0.273,\ 0.342,\ 0.409)$	$(0.272,\ 0.363,\ 0.414)$
$\gimel _{3}$	$(0.354,\ 0.485,\ 0.406)$	$(0.349,\ 0.500,\ 0.408)$
$\gimel _{4}$	$(0.442,\ 0.571,\ 0.354)$	$(0.440,\ 0.573,\ 0.355)$

Step 4(c(i)): Table 9 simplifies the weighted value D-Mx of hybrid averaging by definition 2.6, whereas Table 8 uses hybrid averaging to weighted values.

**Table 8:** Weighted value D-Mx using HA.

$\alpha _{\sigma _{ij}}$	$\digamma _{1}$	$\digamma _{2}$	$\digamma _{3}$	$\digamma _{4}$
$\gimel _{1}$	$(0.156,\ 0.746,\ 0.719)$	$(0.680,\ 0.033,\ -0.151)$	$(0.323,\ 0.668,\ 0.464)$	$(0.206,\ 0.510,\ 0.420)$
$\gimel _{2}$	$(0.132,\ 0.655,\ 0.627)$	$(0.520,\ 0.273,\ 0.113)$	$(0.223,\ 0.346,\ 0.537)$	$(0.192,\ 0.556,\ 0.469)$
$\gimel _{3}$	$(0.153,\ 0.685,\ 0.671)$	$(0.657,\ 0.149,\ 0.018)$	$(0.169,\ 0.769,\ 0.512)$	$(0.330,\ 0.528,\ 0.527)$
$\gimel _{4}$	$(0.231,\ 0.750,\ 0.645)$	$(0.635,\ 0.271,\ 0.009)$	$(0.350,\ 0.712,\ 0.438)$	$(0.386,\ 0.640,\ 0.474)$

**Table 9:** Weighted value D-Mx using HA by applying T-Norm and T-Conorm conditions.

$\alpha _{\sigma _{ij}}$	$\digamma _{1}$	$\digamma _{2}$	$\digamma _{3}$	$\digamma _{4}$
$\gimel _{1}$	$(0.156,\ 0.746,\ 0.719)$	$(0.680,\ 0.033,\ 0.000)$	$(0.323,\ 0.668,\ 0.464)$	$(0.206,\ 0.510,\ 0.420)$
$\gimel _{2}$	$(0.132,\ 0.655,\ 0.627)$	$(0.520,\ 0.273,\ 0.113)$	$(0.223,\ 0.346,\ 0.537)$	$(0.192,\ 0.556,\ 0.469)$
$\gimel _{3}$	$(0.153,\ 0.685,\ 0.671)$	$(0.657,\ 0.149,\ 0.018)$	$(0.169,\ 0.769,\ 0.512)$	$(0.330,\ 0.528,\ 0.527)$
$\gimel _{4}$	$(0.231,\ 0.750,\ 0.645)$	$(0.635,\ 0.271,\ 0.009)$	$(0.350,\ 0.712,\ 0.438)$	$(0.386,\ 0.640,\ 0.474)$

Step 4 (c (ii)): Re-Ordered weighted value hybrid averaging D-Mx is presented in Table 10.

**Table 10:** Reordered weighted value D-Mx using HA.

$\alpha _{\sigma _{ij}}$	$\digamma _{1}$	$\digamma _{2}$	$\digamma _{3}$	$\digamma _{4}$
$\gimel _{1}$	$(0.680,\ 0.033,\ 0.000)$	$(0.206,\ 0.510,\ 0.420)$	$(0.323,\ 0.668,\ 0.464)$	$(0.156,\ 0.746,\ 0.719)$
$\gimel _{2}$	$(0.520,\ 0.273,\ 0.113)$	$(0.223,\ 0.346,\ 0.537)$	$(0.192,\ 0.556,\ 0.469)$	$(0.132,\ 0.655,\ 0.627)$
$\gimel _{3}$	$(0.657,\ 0.149,\ 0.018)$	$(0.330,\ 0.528,\ 0.527)$	$(0.169,\ 0.769,\ 0.512)$	$(0.153,\ 0.685,\ 0.671)$
$\gimel _{4}$	$(0.635,\ 0.271,\ 0.009)$	$(0.386,\ 0.640,\ 0.474)$	$(0.350,\ 0.712,\ 0.438)$	$(0.231,\ 0.750,\ 0.645)$

Step 4(b(iii)): Utilizing the $INHWA_{SW}$ operator presented in Table 11, integrate the qualities for every possibility.

**Table 11:** using $$INHWA_{SW} operator$$.

$\alpha _{\sigma _{i}}$	$\digamma _{j}$
$\gimel _{1}$	$(0.304,\ 0.524,\ 0.437)$
$\gimel _{2}$	$(0.235,\ 0.484,\ 0.470)$
$\gimel _{3}$	$(0.291,\ 0.563,\ 0.467)$
$\gimel _{4}$	$(0.369,\ 0.627,\ 0.426)$

Step 4(d(i)): Hybrid Geometric Weighted Value D-Mx Simplify the weighted value D-Mx of hybrid geometric by definition 2.6 in Tables 12 and 13.

**Table 12:** Weighted value D-Mx using HG.

$\alpha _{\sigma _{ij}}$	$\digamma _{1}$	$\digamma _{2}$	$\digamma _{3}$	$\digamma _{4}$
$\gimel _{1}$	$(0.701,\ 0.196,\ 0.172)$	$(0.029,\ 0.685,\ 0.414)$	$(0.490,\ 0.500,\ 0.299)$	$(0.364,\ 0.342,\ 0.257)$
$\gimel _{2}$	$(0.671,\ 0.119,\ 0.099)$	$(-0.086,\ 0.932,\ 0.777)$	$(0.383,\ 0.190,\ 0.369)$	$(0.349,\ 0.387,\ 0.303)$
$\gimel _{3}$	$(0.697,\ 0.143,\ 0.131)$	$(0.011,\ 0.816,\ 0.666)$	$(0.323,\ 0.608,\ 0.344)$	$(0.497,\ 0.360,\ 0.358)$
$\gimel _{4}$	$(0.782,\ 0.200,\ 0.112)$	$(-0.006,\ 0.930,\ 0.655)$	$(0.518,\ 0.546,\ 0.273)$	$(0.555,\ 0.471,\ 0.307)$

**Table 13:** Weighted value D-Mx using HG by using T-Norm and T-Conorm conditions.

$\alpha _{\sigma _{ij}}$	$\digamma _{1}$	$\digamma _{2}$	$\digamma _{3}$	$\digamma _{4}$
$\gimel _{1}$	$(0.701,\ 0.196,\ 0.172)$	$(0.029,\ 0.685,\ 0.414)$	$(0.490,\ 0.500,\ 0.299)$	$(0.364,\ 0.342,\ 0.257)$
$\gimel _{2}$	$(0.671,\ 0.119,\ 0.099)$	$(0.000,\ 0.932,\ 0.777)$	$(0.383,\ 0.190,\ 0.369)$	$(0.349,\ 0.387,\ 0.303)$
$\gimel _{3}$	$(0.697,\ 0.143,\ 0.131)$	$(0.011,\ 0.816,\ 0.666)$	$(0.323,\ 0.608,\ 0.344)$	$(0.497,\ 0.360,\ 0.358)$
$\gimel _{4}$	$(0.782,\ 0.200,\ 0.112)$	$(0.000,\ 0.930,\ 0.655)$	$(0.518,\ 0.546,\ 0.273)$	$(0.555,\ 0.471,\ 0.307)$

Step 4(d(ii)): Weighted value in a new order Hybrid Geometric D-Mx in Table 14

**Table 14:** Re-Ordered Weighted value D-Mx using HG.

$\alpha _{\sigma _{ij}}$	$\digamma _{1}$	$\digamma _{2}$	$\digamma _{3}$	$\digamma _{4}$
$\gimel _{1}$	$(0.701,\ 0.196,\ 0.172)$	$(0.364,\ 0.342,\ 0.257)$	$(0.490,\ 0.500,\ 0.299)$	$(0.029,\ 0.685,\ 0.414)$
$\gimel _{2}$	$(0.671,\ 0.119,\ 0.099)$	$(0.383,\ 0.190,\ 0.369)$	$(0.349,\ 0.387,\ 0.303)$	$(0.000,\ 0.932,\ 0.777)$
$\gimel _{3}$	$(0.697,\ 0.143,\ 0.131)$	$(0.497,\ 0.360,\ 0.358)$	$(0.323,\ 0.608,\ 0.344)$	$(0.011,\ 0.816,\ 0.666)$
$\gimel _{4}$	$(0.782,\ 0.200,\ 0.112)$	$(0.555,\ 0.471,\ 0.307)$	$(0.518,\ 0.546,\ 0.273)$	$(0.000,\ 0.930,\ 0.655)$

Step 4 (d (iii)): Using the operator $INHWG_{SW}$ in Table 15, integrate the properties for every possibility.

**Table 15:** using $$INHWG_{SW} operator$$.

$\alpha _{\sigma _{i}}$	$\digamma _{j}$
$\gimel _{1}$	$(0.291,\ 0.495,\ 0.313)$
$\gimel _{2}$	$(0.248,\ 0.554,\ 0.487)$
$\gimel _{3}$	$(0.273,\ 0.580,\ 0.446)$
$\gimel _{4}$	$(0.329,\ 0.650,\ 0.411)$

Step 5: Using the definition in 2.5 that is shown in Table 16, determine the ratings for each alternative.

**Table 16:** Scoring and ranking of all AgO.

$\alpha _{\sigma _{i}}$	$\zeta (\gimel _{1})$	$\zeta (\gimel _{2})$	$\zeta (\gimel _{3})$	$\zeta (\gimel _{4})$	Ranking
$INWA_{SW}$ Operator	0.5460	0.4729	0.4849	0.4944	$\zeta (\gimel _{1})>\zeta (\gimel _{4})>\zeta (\gimel _{3})>\zeta (\gimel _{2})$
$INWG_{SW}$ Operator	0.5410	0.4642	0.4783	0.4920	$\zeta (\gimel _{1})>\zeta (\gimel _{4})>\zeta (\gimel _{3})>\zeta (\gimel _{2})$
$INOWA_{SW}$ Operator	0.5122	0.5071	0.4875	0.5059	$\zeta (\gimel _{1})>\zeta (\gimel _{2})>\zeta (\gimel _{4})>\zeta (\gimel _{3})$
$INOWG_{SW}$ Operator	0.5083	0.4985	0.4803	0.5039	$\zeta (\gimel _{1})>\zeta (\gimel _{4})>\zeta (\gimel _{2})>\zeta (\gimel _{3})$
$INHWA_{SW}$ Operator	0.4476	0.4271	0.4205	0.4385	$\zeta (\gimel _{1})>\zeta (\gimel _{4})>\zeta (\gimel _{2})>\zeta (\gimel _{3})$
$INHWG_{SW}$ Operator	0.4940	0.4022	0.4159	0.4230	$\zeta (\gimel _{1})>\zeta (\gimel _{4})>\zeta (\gimel _{3})>\zeta (\gimel _{2})$

Step 6: Sort all options in the order indicated by Table 16.

The findings of the numerical modeling prove the usefulness of the suggested INS DM model to solve the complex issue of pipeline monitoring and maintenance. The model is used to assess the technological options, like quantum-enhanced federated learning to drone swarms, AI-powered repair drones (self-healing pipelines), digital twin-enabled pipelines with real-time drone feedback, and hybrid underwater-aerial drone swarms to pipelines under the circumstances of uncertainty, indeterminacy, and imprecision to select the most reliable option. Quantum Enhanced Federated Learning of Drone Swarms, also referred to as the $\gimel _{1}$ in the case, becomes the choice of action. The technique offers a better insight into the infrastructure resilience, operational reliability, and security needs, and, therefore, enables engineers, energy operators, and policymakers to create potent and flexible frameworks to protect essential energy resources. These insights will be of great use to stakeholders who aim to develop certain interventions for detecting leakage, predictive maintenance, and operations with cyber-resilience. All the applied operators, such as

**Fig. 2:**
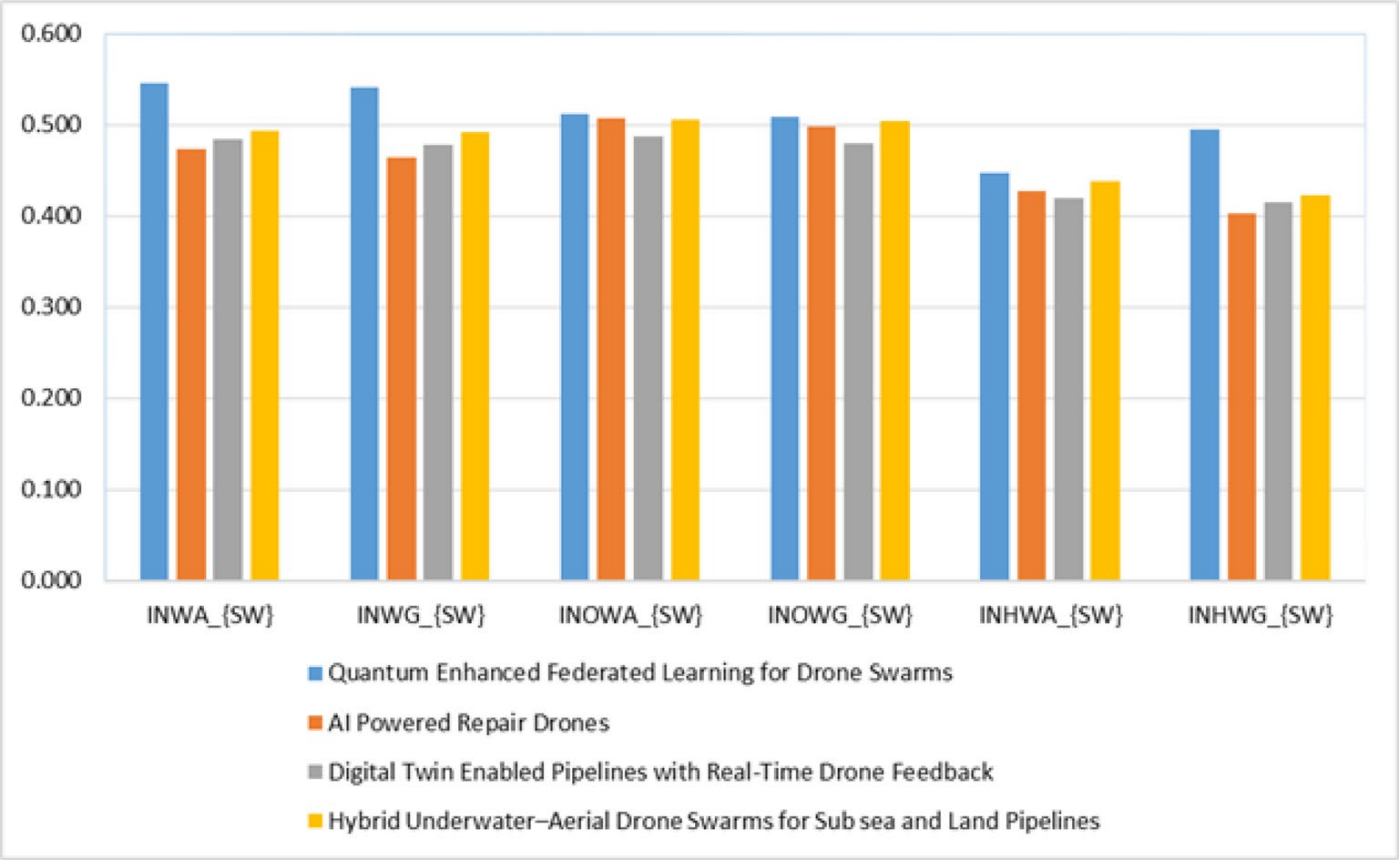
Representation of ranking.

$INWA_{SW}, INWG_{SW}, INOWA_{SW}, INOWG_{SW}, INHWA_{SW}$ and $INHWG_{SW}$ are graphed in Fig. 2.

## Comparison analysis

Here, we bring out the contrast between the suggested INWA operator and the traditional WASPAS system in terms of how the formulated methodology increases DM in the face of federated Edge-AI-supported autonomous drone swarms in detecting natural gas leakage in real-time in natural gas pipelines. Instead of numerical comparison, emphasis is placed on explaining how the peculiarities of the INWA operator offer better flexibility, robustness, and adaptability when compared to the weighted aggregated sum product approach of evaluation. The study allows showing the practical benefits of the proposed operator in addressing complex DM issues by creating a systematic comparison through the prism of complex DM problems and utilizing the multi-criteria and uncertainty factors inherent in the real-life leak detection situation. The suggested INS-Sugeno-Weber framework is compared to the INS-WASPAS approach in order to assess the consistency of decisions, as well as their robustness. WASPAS is chosen as a benchmark because it has a hybrid additive- multiplicative form and it has been extensively used in neutrosophic decision-making studies. The data of numeric ranking and the rank correlation analysis depict that the suggested framework is more stable when there is a growing indeterminacy and a clashing criteria evaluation. The nonlinear nature of aggregation of the Sugeno-Weber norm enables the model to address dominance effect of linear aggregation resulting in more credible rankings in dynamic sensing environments.

**Figure:**
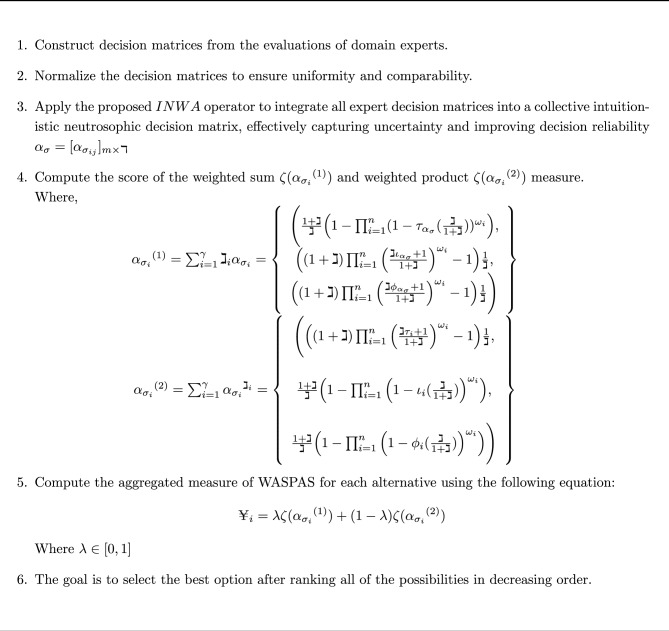
Algorithm

### Numerical illustration

Step 1: Tables 1, 2 and 3 display the DMx from Experts $\yen _{1}$, $\yen _{2}$, and $\yen _{3}$.
Step 2: Due to the benefit-type nature of the characteristics, normalization is not necessary.
Step 3: Utilizing the $INWA_{SW}$ operator, combine all of the INDM in Table 4 to create a community IN DMx.
Step 4-5: Table 17 displays the outcomes obtained by implementing Steps 3-5. These results include the score values of the WSM and the WPM, signified as $\zeta ({\alpha _{\sigma _{i}}}^{(1)})$ and $\zeta ({\alpha _{\sigma _{i}}}^{(2)})$, along with their corresponding rankings. Additionally, let $\lambda =0.5$.

**Table 17:** Results of IN-WASPAS method.

Alternatives	$\zeta ({\alpha _{\sigma _{i}}}^{(1)})$	$\zeta ({\alpha _{\sigma _{i}}}^{(2)})$	${\yen _{{i}}}$	Ranking
$\gimel _{1}$	0.5457	0.5411	0.5434	1
$\gimel _{2}$	0.4729	0.4642	0.4686	4
$\gimel _{3}$	0.4849	0.4783	0.4816	3
$\gimel _{4}$	0.4944	0.4921	0.4932	2

## Discussion

**Fig. 3:**
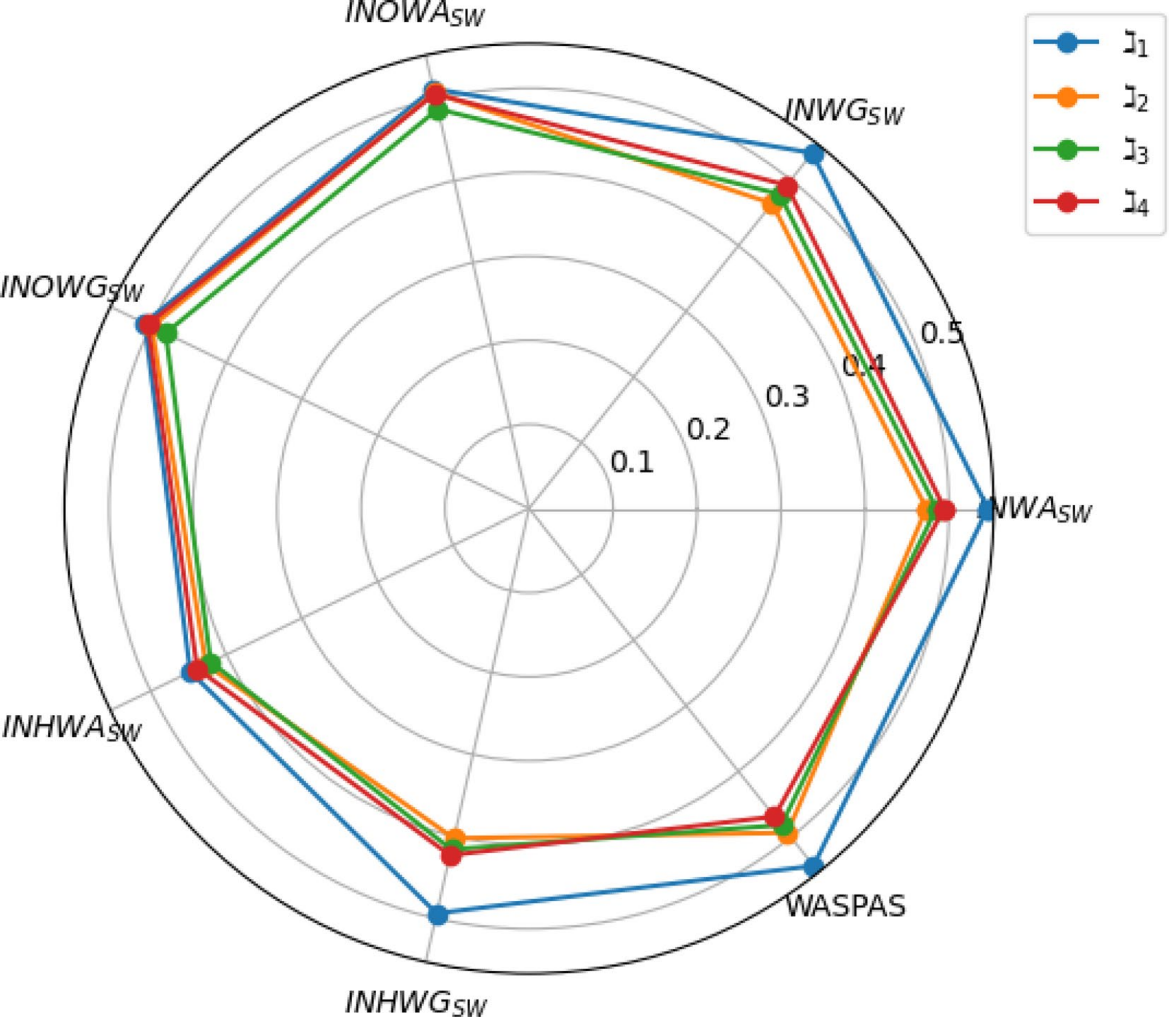
Comparison between $IN_{SW}$, and the WASPAS method.

The INS-based aggregation operator was utilized in this work to check the viability of different options of real-time leak detection in the natural gas pipes by the Federated Edge-AI-enabled autonomous drone swarms. The suggested DM platform, which was established based on INS, offered a systematic way of analyzing the performance of drone swarms in terms of the key indicators, including detection accuracy, reaction time, scalability, adaptiveness to different environments, and its stability to operate in unpredictable regimes. The framework was successful because it takes advantage of the natural complexity and vagueness of the actual leak cases as detected in the real world because INS provides a more global and realistic view of the situation than the traditional ones. One of the most amazing benefits of working with INS is the possibility to manage the components of truth, indeterminacy, and falsity individually and, therefore, minimize the information loss caused by the procedure of aggregation and introduce the picture of the final result of decisions made by the decision-maker into reality. This type of capacity came in especially handy in the experiment with drone swarm solutions in various performance parameters, a vital determinant in natural gas infrastructure inspection, for which erroneous estimations might have purely catastrophic economic, environmental, and safety consequences. The operator was also adaptable enough to also make slight changes to the relations of the criterion to other criteria, like between the detection accuracy and the communication latency, and retain their separate but interconnected quality. To provide the rationale for the appropriateness of the suggested structure, the outcomes of the INS were also juxtaposed against the outcomes of the WASPAS method, which is a well-established MCDM methodology. Numerical and graphical studies in several scenarios had shown that despite certain differentiation dissimilarities of midrange rankings, the two methods invariably produced the same optimal answer. This kind of orientation repeated the efficacy and reliability of the INS operator to operate in complex, uncertainty-based decision situations. The INS frame is more flexible than WASPAS, where the additive and multiplicative processes of aggregation are utilized, because fuzziness and interdependencies that are ignored in other traditional systems are explicitly modeled. The findings of the present study are beneficial to pipeline operators, policymakers, and the advancement of technologies. It should be mentioned that the analysis revealed that both detection accuracy and swarm coordination development can be utilized to mitigate operational risk similarly; thus, strategic investment in either of the two areas can significantly contribute to the reliability of leak detection. In addition, the elements of cost-effectiveness and power efficiency also emerged as one of the factors used to determine the scalability and sustainability of drone swarm applications. Overall, the paper has shown that the concept of Federated Edge-AI application and the use of INS-based aggregation operators to identify leakage in natural gas pipelines in real-time are practical. Beyond this application, there is a high adaptability capacity of the proposed DM framework to other important infrastructure monitoring domains, e.g., smart grids, disaster management, and city safety fundamentals, where the capacity to execute powerful and understandable DM amidst the uncertainty setting is a fundamental requirement (Table 18). It is evident in the experimental comparison between INS-SW and the standard methods like WASPAS that the consistency of the ranking in uncertainty, the better it is, the more reliable the leak detection systems can be. Practically, a stronger and understandable ranking minimizes the false alarms, gives priority to high-risk leakages that should be addressed instantly, and enhance confidence in automated decisions, which are important in the context of pipeline monitoring in real-life. Therefore, the dominance of INS-SW in the benchmarks of MCDM justifies its ability to enhance accuracy of detection and responsiveness across system-level in federated edge-AI systems (Fig. 3).

**Table 18:** Comparison ranking of the proposed SW AgOs with the WASPAS method.

Sr.	Operators	Scoring
1	$INWA_{SW}$ Operator	$\zeta (\gimel _{1})>\zeta (\gimel _{4})>\zeta (\gimel _{3})>\zeta (\gimel _{2})$
2	$INWG_{SW}$ Operator	$\zeta (\gimel _{1})>\zeta (\gimel _{4})>\zeta (\gimel _{3})>\zeta (\gimel _{2})$
3	$INOWA_{SW}$ Operator	$\zeta (\gimel _{1})>\zeta (\gimel _{2})>\zeta (\gimel _{4})>\zeta (\gimel _{3})$
4	$INOWG_{SW}$ Operator	$\zeta (\gimel _{1})>\zeta (\gimel _{4})>\zeta (\gimel _{2})>\zeta (\gimel _{3})$
5	$INHWA_{SW}$ Operator	$\zeta (\gimel _{1})>\zeta (\gimel _{4})>\zeta (\gimel _{2})>\zeta (\gimel _{3})$
6	$INHWG_{SW}$ Operator	$\zeta (\gimel _{1})>\zeta (\gimel _{4})>\zeta (\gimel _{3})>\zeta (\gimel _{2})$
7	WASPAS method	$\gimel _{1}>\gimel _{2}>\gimel _{3}>\gimel _{4}$

## Conclusion

The proposed novel DM method in this study relied on the INS-AgO operator on the enhanced SW norm to assess Federated Edge-AI-enabled autonomous swarms of drones to conduct real-time leak detection in natural gas pipelines. The fact that the SW norm is tunable and the fact that INS is a multidimensional approach were combined in the study to establish a flexible and powerful methodology to address the heterogeneous, unpredictable, and mutually supportive nature of the real-world pipeline monitoring environment. The outcomes of this integration enabled the discovery of complex interrelations between criteria, and it is a respectable demonstration of the operational intricacy of large-scale self-drone deployments. The suggested neutrosophic Sugeno-Weber model of intuitionism has a great potential of making uncertainty-aware decisions in federated drone swarms based on edge-AI. The positive outcomes in numbers prove the superiority in robustness and ranking stability over traditional approaches. In spite of these benefits, there are still practical deployment issues like the computational overhead of edge devices, delay in communication and scalability with large swarms of drones. Comparing various drone swarm plans with several critical operational and infrastructural characteristics has shown how the INS-based framework can be effectively used to address the dependence and uncertainty issues that cannot be adequately considered by the traditional models. The new SW norm added one more parameter of control that gave better control over interactions between criteria, which made the model sensitively responsive to the minor changes in the performance indicators, environmental constraints, and system-level dependencies. This flexibility is important particularly in the modeling of dynamic factors of operation, e.g., heterogeneity of sensors, communication delay, and topographical variability of the pipeline. The mechanism accrual mechanism, which is included in the offered INS system, minimized the information loss, and the strengths and weaknesses of each option were presented clearly and precisely. This complete representation provided the stakeholders with the clear viewpoint of the trade-offs between competing deployment plans and assisted them in making judgments in more informed DM in highly important regions such as investment prioritization, infrastructure upgrades, and technology selection. The efficiency and the reliability of the INS-AgO operator have been verified through the comparative analysis with the WASPAS method at the SW norm. Despite the similarity in the results of the two methods, the INS-based model was more sensitive in the context of identifying intermediary variation and situations with context-specific interactions, which is a key feature of difficult situations of decision-making when criteria are relative to each other as well as dynamically changing. The results demonstrate that the INS model is a more precise and situation-specific evaluation in comparison with the conventional MCDM methods. Overall, the findings show that the SW-enhanced INS model is well-grounded in theory and can be applied to practice. It fills the void between the abstract DM theory and the concrete task, i.e., the scalable and versatile decision-support system, the one that is perfectly suited in such areas as autonomous drone swarming, uncertainty, interdependence, and responsiveness, which are important. This can be used in the scenario of natural gas infrastructure as a structured and credible method of optimization of the swarm of drone programs in the ambiguous, dynamic, and complex operational settings. Any of the avenues can be extended in the future by connecting blockchain to the safe data exchange, digital twins to the predictive maintenance, and cutting-edge edge-AI to the real-time data analytic. The comparative analysis to the other state-of-the-art MCDM tools like MABAC, CoCoSo, and temporal and regional analysis can be potentially useful in achieving a better insight into the influences of the environmental and operational conditions on long-term strategic performance. Such developments would allow the applicability, validity, and external validity of the INS model to a vast region of the land where uncertainty and criterion interdependency are the factors that comprise successful DM.

## Data Availability

No datasets were generated or analysed during the current study.
